# Supplementary material for: Pharmacokinetics and Metabolomic Profiling of Metformin and Andrographis paniculata: A Protocol for a Crossover Randomised Controlled Trial
Source: J Clin Med. 2022 Jul 6;11(14):3931. doi: 10.3390/jcm11143931 (PMC9323336; doi:10.3390/jcm11143931)
Supplement: Supplementary file 1 [file jcm-11-03931-s001.zip › Document S1. Clinical Trial Protocol P1-PKPD-Metabolomics V4.0 170919.pdf]

TITLE:

A Phase 1, Open Label, Randomized, Three-Period, Crossover, Single Dose Oral Administration Of *Andrographis Paniculata* And Metformin Clinical Trial In Healthy Volunteers Under Fasting Condition.

Protocol Number : P1-PKPD-Metabolomic

Version : 4.0

Date : 17 September 2019

SUPERVISOR:

DR. LUQMAN BIN IBRAHIM
PROFESOR MADYA DR. HASNIZA BINTI ZAMAN HURI
PROFESOR MADYA DR. NAJIHAH BINTI MOHD HASHIM

STUDY SITE:

Clinical Investigation Center,

University Malaya Medical Center,

Lembah Pantai,

59100 Kuala Lumpur.

Table of Contents

ABBREVIATION 5

1.0 BACKGROUND INFORMATION/ RATIONAL FOR THE STUDY: 6

1.1 Background information of this research 6

1.2 Rational of selection the investigational product 6

2.0 TRIAL OBJECTIVES: 8

3.0 INVESTIGATIONAL PRODUCTS 9

3.1 *Andrographis paniculata* 9

3.1.1 Compound for *Andrographis paniculata* 9

3.1.2 Pharmacological effects 9

3.1.3 Contraindications 12

3.1.4 Precautions 13

3.1.5 Adverse reactions 13

3.1.6 Dosage and administration 13

3.2 Metformin 13

3.2.1 Chemical compound 13

3.2.2 Clinical Pharmacology 13

3.2.3 Pharmacokinetics 14

3.2.4 Contraindications 14

3.2.6 Adverse Reactions 17

3.2.7 Dosage and administration 17

4.0 INVESTIGATIONAL PLAN 17

4.1 Overall Study Design: 17

4.2 Discussion of study design 17

4.3 Benefit-Risk Evaluation 19

4.4 Selection of Study Population 19

4.4.1 Inclusion Criteria: 19

4.4.2 Exclusion Criteria: 19

4.4.3 Withdrawal Criteria 20

4.5 Study Medication 20

4.5.1 Identity of Investigational Products 20

4.5.2 Receipt, Storage and Retention 21

4.5.3 Method of Assigning Volunteers to Treatment Group 21

4.5.4 Randomization and Blinding 22

4.5.5 Drug Accountability 22
4.5.6 Prior and Concomitant Medication 22
5.0 STUDY PROCEDURES 22
5.1 Pre-Study Procedures 22
5.1.1 Demography Information 22
5.1.2 12-Leas Electrocardiogram 22
5.1.3 Medical Assessment of volunteers 22
5.1.4 Vital Signs 22
5.1.5 Laboratory Tests 23
5.2 Planned Activity during the study 23
5.2.1 Enrolment 23
5.2.2 Housing 23
5.2.3 Restrictions 23
5.2.4 Drug Administration 24
5.2.5 Diet 24
5.2.6 Blood sampling for drug analysis 25
5.3 Adverse Events 26
5.3.1 Definitions 26
5.3.2 Severity of adverse events 27
5.3.3 Relationship to the Study Drugs 27
5.3.4 Reporting and Documentation of Adverse Events 28
5.3.5 Emergency Procedures 28
5.4 Post-study Procedures 28
5.4.1 Discharge from CIC ward 28
5.4.2 Follow up 28
5.5 Volunteer Replacement 28
6.0 SAFETY ASSESSMENT 29
6.1 During Study Vital Sings 29
6.2 Physical Examination 29
6.3 Handling and Reporting of Adverse Event 29
7.0 ANALYSIS 29
8.0 PHARMACOKINETIC EVALUATION 30
8.1 Pharmacokinetic Parameters 30
8.2 Treatment of Time Deviation 30

9.0 STATISTICAL METHODS 30
9.1 Concentration-time profile and pharmacokinetic parameters 30
9.2 Multivariate analysis 30
9.3 Handling of Dropouts or Missing Data 30
9.4 Pre-dose Concentration 31
9.5 Data Due to Vomiting 31
10.0 STUDY DOCUMENTATION 31
10.1 Final report 31
10.2 Deviation from Protocol 31
10.3 Archiving 31
11.0 ETHICAL AND LEGAL ASPECTS OF THE CONDUCT OF STUDY 31
11.1 Ethics 31
11.2 Regulatory Requirements 32
12.0 PUBLICATION AND PRESENTATION 32
13.0 SUBJECT CONFIDENTIALITY 32
14.0 MEDICAL CARE AND TREATMENT FOLLOWING STUDY COMPLETION 32
15.0 APPENDICES 33
15.1 Appendix I: Declaration of Helsinki (WMA, 2013) 33
16.0 REFERENCES: 40

ABBREVIATION

ADR	Adverse Drug Reaction
AE	Adverse Event
ALP	Alkaline Phosphatase
ALT	Alanine Aminotransferase
AST	Aspartate Aminotransferase
AUC	Area under the plasma concentration
BMI	Body Mass Index
BP	Blood Pressure
Cmax	Maximum plasma concentration
Conc.	Concentration
CRF	Case Report Form
CV	Coefficient of Variation
EC	Ethics Committee
ECG	Electrocardiogram
FBC	Full Blood Count
GCP	Good Clinical Practice
IB	Investigator Brochure
ICF	Informed Consent Form
IP	Investigational Products
LFT	Liver Function Test
PK	Pharmacokinetic
RA	Regulatory Authority
RBS	Random blood sugar
SAE	Serious Adverse Events
T1/2	Time taken by plasma concentration to reduce to 50% during the elimination phase
Tmax	Time required to reach maximum concentration of drug in plasma

1.0 BACKGROUND INFORMATION/ RATIONAL FOR THE STUDY:

1.1 Background information of this research

According to the guidance on General Consideration for Clinical Trials ICH E8, phase 1 clinical trial typically involved one or a combination of the following aspects:

(a) Estimation of initial safety and tolerability
(b) Pharmacokinetics
(c) Assessment of pharmacodynamics
(d) Early measurement of drug activity

Traditional herbal medicines have been used for decades around the world and there are safe as home remedial. There are various non-clinical pharmacology and toxicology studies performed for herbal medicine. However, identification of the lead compound or biomarker from the herbal medicine remains a huge challenge to the researcher.

Most of the traditional herbal medicine could not provide evidence of bioavailability in drug development process. Kantae et al proposed that discovery of metabolite biomarkers using pharmacometabolomics could predict the pharmacokinetic and pharmacodynamic effects (Kantae et al., 2017). Several nonclinical studies and clinical trials had been performed to explore the pharmacokinetics and pharmacodynamic effects for herbal medicine (Hao et al., 2015, Wu et al., 2015, Miao and Wang, 2013, Sheridan et al., 2012).

In this study, I would like to propose that pharmacometabolomic study could predict the bioavailability and dose relation of herbal medicine and known medicinal product with the relevant biomarkers and metabolites after single dose oral administration.

1.2 Rational of selection the investigational product

Andrographis paniculata or hempedu bumi capsule is a traditional medicinal product registered under Ministry of Health for general wealth being. There are several studies that has been conducted for *Andrographis paniculata.*

Study	Design	Treatment	Max dose/ day	n
(Agarwal et al., 2005)	Open label, parallel design in type 2 diabetes mellitus	*Andrographis paniculata* 600mg bd for 2 weeks<br>Increase the dose 300mg everytime for 8 weeks<br>900mg bd for 2 weeks	1800mg *Andrographis paniculata*<br>Each capsule contained 5.45% andrographolide	20
(Sandborn et al., 2013)	Randomized, double-blind, placebo-controlled trial in	*A paniculata* extract for 8 weeks	1200mg and 1800mg daily	45

	mild-to-moderate ulcerative colitis			
(Melchior et al., 2000)	Phase 3, randomized, double-blind, placebo-controlled parallel group clinical trial for uncomplicated upper-respiratory tract infections	Kan Jang tablets consist of Andrographis paniculata and Acanthopanax senticosus<br><br>4 tablets 3 times a day (approximately 60mg andrographolide) for 3-6 days. 1 tablet containing 5.25 andrographolide.	approximately 60mg andrographolide / day	46 pilot study<br><br>179 for phase 3

However, the absorption of andrographolide was only measured by a study using mixed herbals formulation. Here, I would like to explore the rate and extend of absorption for andrographolide into human blood circulation using targeted approach and also identify the secondary metabolites using untargeted metabolomics approach.

The rational of choosing *Andrographis paniculata* 1000mg and 2000mg is based on the approved dose of capsule *Andrographis paniculata* for traditional use. The approved dose is 2 capsules 2 times a day.

2.0 TRIAL OBJECTIVES:

Objective:

1. To assess the bioavailability of *Andrographis paniculata* 1000mg and 2000mg capsules after single dose of oral administration in healthy human volunteers under fasting condition.
2. To assess the bioavailability of Metformin 1000mg after single dose of oral administration in healthy human volunteers under fasting condition.

Secondary objectives:

1. To determine the pharmacodynamic effects of *Andrographis paniculata* using metabolomics approach.
2. To evaluate the safety of *Andrographis paniculata* 1000mg and 2000mgs capsules after single dose of oral administration in healthy human volunteers under fasting condition.
3. To determine the pharmacodynamic effects of Metformin using metabolomics approach.
4. To evaluate the safety of Metformin after single dose of oral administration in healthy human volunteers under fasting condition.

Primary endpoint :

1. To calculate the Cmax, AUC, T1/2 and Tmax of andrographolide being absorbed after single dose of *Andrographis paniculata* 1000mg and 2000mg oral administration by healthy human volunteers under fasting condition
2. To calculate the Cmax, AUC, T1/2 and Tmax of Metformin being absorbed after single dose of Metformin 1000mg oral administration by healthy human volunteers under fasting condition

Secondary endpoint :

1. To determine the secondary metabolites of drug compounds from *Andrographis paniculata* capsules oral administration and draw the relevant metabolic pathway using heatmap, dendrogram, mummichog diagram and 3-dimensional network visual diagram.
2. To identify the adverse effects of oral administration of *Andrographis paniculata* capsules.
3. To determine the secondary metabolites of drug compounds from Metformin oral administration and draw the relevant metabolic pathway using heatmap, dendrogram, mummichog diagram and 3-dimensional network visual diagram.
4. To identify the adverse effects of oral administration of Metformin tablet.

3 .0 INVESTIGATIONAL PRODUCTS

3.1 *Andrographis paniculata*

3.1.1 Compound for *Andrographis paniculata*

Capsule *Andrographis paniculata* also known as King of Bitter, Hempedu bumi, Chuan Xin Lian and Karmegh. The major compounds extracted from *Andrographis paniculata* includes andrographolide, 11,12-didehydro-14-deoxyandrographolide, andropanoside (*1*, *3*, *6*, *7*, *9*, *16*), deoxyandrographolide, andrographiside, neoandrographolide and deoxyandrographiside. The structure of andrographolide is as below:

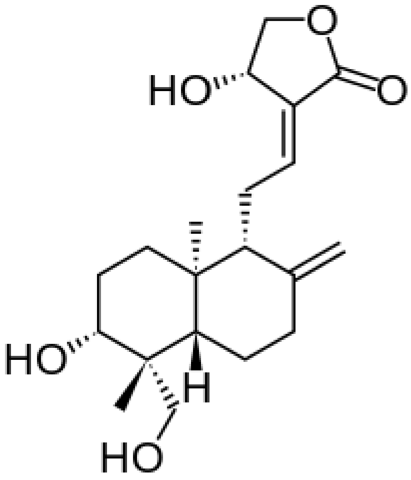

Figure 1 Structure for Andrographolide

The main compound in the *Andrographis paniculata* is andrographolide. Andrographolide also known as xiyanping in China. In China, xiyangping injection is marketed as a traditional medicinal product for injection.

3.1.2 Pharmacological effects

Antibacterial activity

An ethanol extract of the leaves inhibited the growth in vitro of *Escherichia coli* (Foo et al., 2018) and *Staphylococcus aureus*. A 50% methanol extract of the leaves inhibited growth in vitro of *Proteus vulgaris*. However, no in vitro antibacterial activity was observed when dried powder from the aerial parts was tested against *E. coli*, *Staphylococcus aureus*, *Salmonella typhi* or *Shigella* species.

Immunostimulatory activity

Intragastric administration of an ethanol extract of the aerial parts (25mg/kg body weight) or purified andrographolides (1 mg/kg body weight) to mice stimulated antibody production and the delayed-type hypersensitivity response to sheep red blood cells. The extract also stimulated a non-specific immune response in mice, measured by macrophage migration index, phagocytosis of [^{14}C]leucine-labelled *E. coli*, and proliferation of splenic lymphocytes. The extract was more effective than

either andrographolide or neoandrographolide alone, suggesting that other constituents may be involved in the immunostimulant response.

Antipyretic activity

Intragastric administration of an ethanol extract of the aerial parts (500mg/kg body weight) to rats decreased yeast-induced pyrexia. The extract was reported to be as effective as 200 mg/kg body weight of aspirin, and no toxicity was observed at doses up to 600 mg/kg body weight. Intragastric administration of andrographolide (100 mg/kg body weight) to mice decreased brewer's yeast-induced pyrexia. Intragastric administration of deoxyandrographolide, andrographolide, neoandrographolide or 11,12-didehydro-14-deoxyandrographolide (100 mg/kg body weight) to mice, rats or rabbits reduced pyrexia induced by 2,4-dinitrophenol or endotoxins.

Antidiarrhoeal activity

Herbal Andrographidis has antidiarrhoeal activity in situ. An ethanol, chloroform or 1-butanol extract of the aerial parts (300mg/ml) inhibited the *E. coli* enterotoxin-induced secretory response - which causes a diarrhoeal syndrome - in the rabbit and guinea-pig ileal loop assay. However, an aqueous extract of the aerial parts was not active. The constituent diterpene lactones, andrographolide and neoandrographolide, exhibited potent antisecretory activity in vivo against *E. coli* enterotoxin-induced diarrhoea. Andrographolide (1 mg per loop) was as active as loperamide when tested against heat-labile *E. coli*enterotoxin-induced diarrhoea and more effective than loperamide when tested against heat-stable *E. coli* enterotoxin-induced diarrhoea. Neoandrographolide (1 mg per loop) was as effective as loperamide when tested against heat-labile *E. coli*enterotoxin-induced diarrhoea and slightly less active than loperamide when tested against heat-stable *E. coli* enterotoxin-induced diarrhoea. The mechanism of action involves inhibition of the intestinal secretory response induced by heat-labile *E. coli*enterotoxins, which are known to act through the stimulation of adenylate cyclase, and by inhibition of the secretion induced by heat-stable *E. coli* enterotoxins, which act through the activation of guanylate cyclase (*39*). Incubation of murine macrophages with andrographolide (1-50 µmol/l) inhibited bacterial endotoxin-induced nitrite accumulation in a concentration- and timedependent manner. Western blot analysis demonstrated that andrographolide inhibited the expression of an inducible isoform of nitric oxide synthase linked to endotoxin-induced circulatory shock.

Anti-inflammatory activity

Intragastric administration of deoxyandrographolide, andrographolide, neoandrographolide or 11,12-didehydrodeoxyandrographolide to mice inhibited the increase in cutaneous or peritoneal capillary permeability induced by xylene or acetic acid, and reduced acute exudation in Selye granulocysts treated with croton oil. 11,12-Didehydrodeoxyandrographolide had the most potent antiinflammatory activity in vivo.

Antimalarial activity

A 50% ethanol extract of the aerial parts inhibited the growth of *Plasmodium berghei* both in vitro (100 mg/ml) and in mice after intragastric administration (1 g/kg body weight). Intragastric administration of a 1-butanol, chloroform or ethanol-water extract of the aerial parts to *Mastomys natalensis* inhibited the growth of *P. berghei* at doses of 1-2 g/kg body weight. Andrographolide (5 mg/kg body weight) and neoandrographolide (2.5mg/kg body weight) were also effective when administered by gastric lavage (Najib Nik et al., 1999).

Antihepatotoxic activity

The aerial parts and their constituent andrographolides have antihepatotoxic activity in vitro and in vivo (*45-54*). Intraperitoneal administration of a methanol extract of the aerial parts (861.3 mg/kg body weight) to mice reduced hepatotoxicity induced by carbon tetrachloride (CCl_4), and reversed CCl_4-induced histopathological changes in the liver. Intraperitoneal administration of andrographolide (100 mg/kg body weight) to mice inhibited the CCl_4-induced increase in the activity of serum glutamate oxaloacetate transaminase, serum glutamate pyruvate transaminase, alkaline phosphatase, bilirubin and hepatic triglycerides. Intraperitoneal administration of a methanol extract of the aerial parts (500 mg/kg body weight) to rats also suppressed the CCl_4-induced increase in the activity of serum glutamate oxaloacetate transaminase, serum glutamate pyruvate transaminase, alkaline phosphatase and bilirubin. Intragastric administration of an aqueous extract of the aerial parts (500mg/kg body weight) to ethanol-treated rats decreased the activity of serum transaminases and suppressed histopathological changes in the liver. Andrographolide, the major antihepatotoxic component of the plant, exerted a pronounced protective effect in rats against hepatotoxicity induced by CCl4, Dgalactosamine, paracetamol and ethanol. Andrographolide was more effective than silymarin, the standard hepatoprotective agent.

The common cold

Herbal Andrographidis has been used clinically for symptomatic treatment of the common cold and uncomplicated sinusitis, pharyngotonsillitis, pneumonia and bronchitis. A placebo-controlled, double-blind clinical trial assessed the efficacy of a standardized extract of the aerial parts (containing 4% andrographolides) for treatment of the common cold in 61 adult patients. A significant reduction ($P < 0.0001$) in clinical symptoms such as sore throat, tiredness, muscular ache and malaise was observed on day 4 in the group receiving 1200 mg extract daily, as compared with the placebo group. No adverse reactions were reported in either group.

A randomized, placebo-controlled, double-blind pilot trial was conducted to evaluate the efficacy of a standardized extract of the aerial parts (containing 4% andrographolides) on the initial symptoms of the common cold and uncomplicated sinusitis. Fifty adult patients received either 1020mg extract or a placebo daily for 5 days. The results demonstrated that patients in the treated group took less sick leave than those in the placebo group (0.21 day compared to 0.96 day). Furthermore, 68% of treated patients felt totally recovered, as compared with 36% of the placebo group. Also 55% of the treated patients thought that the course of illness was much easier than normal, as compared with 19% of the placebo group.

A randomized, placebo-controlled, double-blind study evaluated a standardized extract of the aerial parts (containing 4% andrographolides) in the prophylaxis of the common cold in 107 schoolchildren during the winter season. The children received either 200 mg extract or a placebo daily for 3 months and were evaluated weekly by a physician. There was no difference in the occurrence of colds between the two groups during the first 2 months of treatment. However, after the third month of treatment, there was a significant difference ($P < 0.05$) in the occurrence of the common cold in the treated group (30%) as compared with the placebo group (62%).

A randomized, double-blind comparison study of 152 adult patients with pharyngotonsillitis evaluated the efficacy of powdered aerial parts (6 g daily) and paracetamol (1 capsule of 325 mg as needed) for improving symptomatology. Baseline evaluation showed no significant difference between the two groups. The crude drug was as effective as paracetamol in reducing the incidence of sore throat and fever after 3 days of treatment. In a study without controls, treatment of patients with a standardized extract of *A. paniculata* (containing 4% andrographolides) reduced the incidence of fever associated with the common cold. The body temperature of patients treated with the extract was lowered in less than 48 hours after treatment. This finding was con- firmed in a later study.

Urinary infections

A clinical trial compared the efficacy of Herbal Andrographidis, co-trimoxazole (sulfamethoxazole + trimethoprim) and norfloxacin in the prevention of urinary tract infections after extracorporeal shock wave lithotripsy. Patients received a 5-day course of either Herbal Andrographidis (4 tablets of 250mg, three times daily) or co-trimoxazole (2 tablets of 25 mg, twice daily) or norfloxacin (1 tablet of 200 mg, twice daily). After 1 month of treatment, urinalysis results of 100 patients demonstrated that pyuria, haematuria and proteinuria were reduced in all treatment groups, and there was no significant difference between the three treatments.

Dysentery

The aerial parts have been used for the treatment of acute bacillary dysentery and enteritis. In clinical studies, the combination of andrographolide and neoandrographolide was reported to be more effective than either furazolidine or chloramphenicol in the treatment of bacillary dysentery. A randomized, double-blind clinical study of 200 patients compared the efficacy of the powdered aerial parts with tetracycline in the treatment of acute diarrhoea and bacillary dysentery. Patients received capsules of either the aerial parts or tetracycline (both 500 mg, four times daily) for 3 days. Compared with tetracycline, the aerial parts decreased the diarrhoea (both the frequency and amount of discharge). Furthermore, the aerial parts were more effective in treating diarrhoea resulting from shigellosis than from cholera.

3.1.3 Contraindications

Herbal Andrographidis should not be used during pregnancy or lactation.

Herbal Andrographidis is contraindicated in cases of known allergy to plants of the Acanthaceae family.

3.1.4 Precautions

Due to potential anaphylactic reactions, crude extracts of Herbal Andrographidis should not be injected (*6*, *56*).

Drug interactions

Extracts of Herbal Andrographidis may have a synergistic effect with isoniazid (*6*).

3.1.5 Adverse reactions

Large oral doses of Herbal Andrographidis may cause gastric discomfort, vomiting and loss of appetite (*6*). These side-effects appear to be due to the bitter taste of andrographolide (*6*). Anaphylactic reactions may occur if the crude drug extract is injected (*6*, *56*). Two cases of urticaria have been reported (*18*).

3.1.6 Dosage and administration

For pyrexia: a decoction from 3 g crude drug, twice daily (*1*, *5*). For the common cold: 1.5-3.0 g powdered crude drug three times daily, after meals and at bedtime (*1*). For diarrhoea: a decoction from 3-9 g crude drug as a single dose as needed (*1*, *5*), or two tablets of 500 mg four times daily, after meals and at bedtime (*5*).

3.2 Metformin

3.2.1 Chemical compound

The brand name of Metformin is Glucophage. It is an oral glucose-lowering drug of the biguanides class. The chemical name for Metformin is 1,1-dimethylbiguanide hydrochloride or 1,1-dimethylbiguanide hydrochloride. The molecular formula is C4H11N5 and molecular weight is 129.167 g/mol.

The structure formula is:

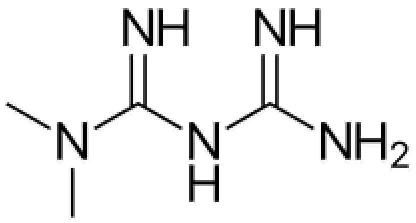

3.2.2 Clinical Pharmacology

Metformin is an antihyperglycemic agent which improves glucose tolerance in patients with type 2 diabetes, lowering both basal and postprandial plasma glucose. Its pharmacologic mechanisms of action are different from other classes of oral antihyperglycemic agents. Metformin decreases hepatic glucose production,

decreases intestinal absorption of glucose, and improves insulin sensitivity by increasing peripheral glucose uptake and utilization. Unlike sulfonylureas, metformin does not produce hypoglycemia in either patients with type 2 diabetes or normal subjects, and does not cause hyperinsulinemia. With metformin therapy, insulin secretion remains unchanged while fasting insulin levels and day-long plasma insulin response may actually decrease.

3.2.3 Pharmacokinetics

Absorption and Bioavailability
The absolute bioavailability of a GLUCOPHAGE 500 mg tablet given under fasting conditions is approximately 50% to 60%. Studies using single oral doses of GLUCOPHAGE 500 to 1500 mg, and 850 to 2550 mg, indicate that there is a lack of dose proportionality with increasing doses, which is due to decreased absorption rather than an alteration in elimination. Food decreases the extent of and slightly delays the absorption of metformin, as shown by approximately a 40% lower mean peak plasma concentration (Cmax), a 25% lower area under the plasma concentration versus time curve (AUC), and a 35-minute prolongation of time to peak plasma concentration (Tmax) following administration of a single 850 mg tablet of metformin with food, compared to the same tablet strength administered fasting. The clinical relevance of these decreases is unknown.

Distribution
The apparent volume of distribution (V/F) of metformin following single oral doses of GLUCOPHAGE 850 mg averaged 654 ± 358 L. Metformin is negligibly bound to plasma proteins, in contrast to sulfonylureas, which are more than 90% protein bound. Metformin partitions into erythrocytes, most likely as a function of time. At usual clinical doses and dosing schedules of GLUCOPHAGE, steady state plasma concentrations of metformin are reached within 24 to 48 hours and are generally

Metabolism and Elimination
Intravenous single-dose studies in normal subjects demonstrate that metformin is excreted unchanged in the urine and does not undergo hepatic metabolism (no metabolites have been identified in humans) nor biliary excretion. Renal clearance (see Table 1) is approximately 3.5 times greater than creatinine clearance, which indicates that tubular secretion is the major route of metformin elimination. Following oral administration, approximately 90% of the absorbed drug is eliminated via the renal route within the first 24 hours, with a plasma elimination half life of approximately 6.2 hours. In blood, the elimination half-life is approximately 17.6 hours, suggesting that the erythrocyte mass may be a compartment of distribution.

3.2.4 Contraindications

Metformin is contraindicated in patients:
1. Severe renal impairment (eGFR below 30 mL/min/1.73 m^2).
2. Known hypersensitivity to metformin hydrochloride.
3. Acute or chronic metabolic acidosis, including diabetic ketoacidosis, with or without coma. Diabetic ketoacidosis should be treated with insulin.

3.2.5 Precautions

Lactic acidosis—There have been postmarketing cases of metformin-associated lactic acidosis, including fatal cases. These cases had a subtle onset and were accompanied by nonspecific symptoms such as malaise, myalgias, abdominal pain, respiratory distress, or increased somnolence; however, hypotension and resistant bradyarrhythmias have occurred with severe acidosis. Metformin-associated lactic acidosis was characterized by elevated blood lactate concentrations (>5 mmol/L), anion gap acidosis (without evidence of ketonuria or ketonemia), and an increased lactate: pyruvate ratio; metformin plasma levels were generally >5 mcg/mL. Metformin decreases liver uptake of lactate increasing lactate blood levels which may increase the risk of lactic acidosis, especially in patients at risk.

If metformin-associated lactic acidosis is suspected, general supportive measures should be instituted promptly in a hospital setting, along with immediate discontinuation of Metformin. In Metformin treated patients with a diagnosis or strong suspicion of lactic acidosis, prompt hemodialysis is recommended to correct the acidosis and remove accumulated metformin (metformin hydrochloride is dialyzable with a clearance of up to 170 mL/min under good hemodynamic conditions). Hemodialysis has often resulted in reversal of symptoms and recovery.

Educate patients and their families about the symptoms of lactic acidosis and, if these symptoms occur, instruct them to discontinue Metformin and report these symptoms to their healthcare provider.

Renal impairment—The postmarketing metformin-associated lactic acidosis cases primarily occurred in patients with significant renal impairment.

The risk of metformin accumulation and metformin-associated lactic acidosis increases with the severity of renal impairment because metformin is substantially excreted by the kidney. Clinical recommendations based upon the patient's renal function include

- Before initiating Metformin, obtain an estimated glomerular filtration rate (eGFR)
- Metformin is contraindicated in patients with an eGFR less than 30 mL/min/1.73m^2.
- Initiation of Metformin is not recommended in patients with eGFR between 30-45 mL/min/1.73 m^2.
- Obtain an eGFR at least annually in all patients taking Metformin. In patients at risk for the development of renal impairment (e.g., the elderly), renal function should be assessed more frequently.
- In patients taking Metformin whose eGFR falls below 45 mL/min/1.73 m 2 , assess the benefit and risk of continuing therapy.

Drug interactions—The concomitant use of GLUCOPHAGE or GLUCOPHAGE XR with specific drugs may increase the risk of metformin-associated lactic acidosis: those that impair renal function, result in significant hemodynamic change, interfere with acid-base balance, or increase metformin accumulation. Consider more frequent monitoring of patients.

Age 65 or greater—The risk of metformin-associated lactic acidosis increases with the patient's age because elderly patients have a greater likelihood of having hepatic, renal, or cardiac impairment than younger patients. Assess renal function more frequently in elderly patients.

Radiologic studies with contrast—Administration of intravascular iodinated contrast agents in metformin-treated patients has led to an acute decrease in renal function and the occurrence of lactic acidosis. Stop Metformin at the time of, or prior to, an iodinated contrast imaging procedure in patients with an eGFR between 30 and 60 mL/min/1.73 m^2; in patients with a history of hepatic impairment, alcoholism or heart failure; or in patients who will be administered intra-arterial iodinated contrast. Re-evaluate eGFR 48 hours after the imaging procedure, and restart Metformin if renal function is stable.

Surgery and other procedures—Withholding of food and fluids during surgical or other procedures may increase the risk for volume depletion, hypotension, and renal impairment. Metformin should be temporarily discontinued while patients have restricted food and fluid intake.

Hypoxic states—Several of the postmarketing cases of metformin-associated lactic acidosis occurred in the setting of acute congestive heart failure (particularly when accompanied by hypoperfusion and hypoxemia). Cardiovascular collapse (shock), acute myocardial infarction, sepsis, and other conditions associated with hypoxemia have been associated with lactic acidosis and may cause prerenal azotemia. When such an event occurs, discontinue Metformin.

Excessive alcohol intake—Alcohol is known to potentiate the effect of metformin on lactate metabolism. Patients, therefore, should be warned against excessive alcohol intake, acute or chronic, while receiving Metformin.

Hepatic impairment—Patients with hepatic impairment have developed cases of metformin associated lactic acidosis. This may be due to impaired lactate clearance resulting in higher lactate blood levels. Therefore, avoid use of Metformin in patients with clinical or laboratory evidence of hepatic disease.

Hypoglycemia—Hypoglycemia does not occur in patients receiving Metformin alone under usual circumstances of use, but could occur when caloric intake is deficient, when strenuous exercise is not compensated by caloric supplementation, or during concomitant use with other glucose-lowering agents (such as sulfonylureas and insulin) or ethanol.

Elderly, debilitated, or malnourished patients, and those with adrenal or pituitary insufficiency or alcohol intoxication are particularly susceptible to hypoglycemic effects. Hypoglycemia may be difficult to recognize in the elderly, and in people who are taking beta-adrenergic blocking drugs.

Macrovascular outcomes—There have been no clinical studies establishing conclusive evidence of macrovascular risk reduction with Metformin or any other antidiabetic drug.

3.2.6 Adverse Reactions

Diarrhea led to discontinuation of study medication in 6% of patients treated with Metformin. Additionally, the following adverse reactions were reported in ≥1.0% to ≤5.0% of Metformin patients and were more commonly reported with Metformin than placebo: abnormal stools, hypoglycemia, myalgia, lightheaded, dyspnea, nail disorder, rash, sweating increased, taste disorder, chest discomfort, chills, flu syndrome, flushing, palpitation.

Cholestatic, hepatocellular, and mixed hepatocellular liver injury have been reported with postmarketing use of metformin.

3.2.7 Dosage and administration

There is no fixed dosage regimen for the management of hyperglycemia in patients with type 2 diabetes with Metformin or any other pharmacologic agent. Dosage of Metformin must be individualized on the basis of both effectiveness and tolerance, while not exceeding the maximum recommended daily doses. The maximum recommended daily dose of Metformin is 2550 mg in adults and 2000 mg in pediatric patients (10-16 years of age).

The usual starting dose of Metformin Tablets is 500 mg twice a day or 850 mg once a day, given with meals. In general, clinically significant responses are not seen at doses below 1500 mg per day. Dosage increases should be made in increments of 500 mg weekly or 850 mg every 2 weeks, up to a total of 2000 mg per day, given in divided doses. The dosage of Metformin must be individualized on the basis of both effectiveness and tolerability. Patients can also be titrated from 500 mg twice a day to 850 mg twice a day after 2 weeks. For 25 Reference ID: 4079189 those patients requiring additional glycemic control, Metformin may be given to a maximum daily dose of 2550 mg per day. Doses above 2000 mg may be better tolerated given 3 times a day with meals.

4.0 INVESTIGATIONAL PLAN

4.1 Overall Study Design:

An open label, randomised, three-periods, cross over study to identify the bioavailability of capsule *Andrographis paniculata (Hempedu bumi)* 1000mg, 2000mg and Metformin 1000mg under fasting condition in healthy volunteers. A wash-out period of at least 7 days is planned between the two periods. Approximately 25 subjects will be screened for eligibility and 18 subjects will be enrolled and dose in the study.

4.2 Discussion of study design

In the process of a drug development, it is important to investigate the bioavailability of the new medicinal product. The efficacy and safety of this drug is well established.

Both medicinal products are registered with Ministry of Healthy Malaysia. This study will be conducted with the aim to investigate whether the rate and extend of absorption exist for the *Andrographis paniculata* 1000mg, 2000mg and Metformin 1000mg.

<u>Choice of Volunteers</u>

Bioavailability is generally recommended to be examined in volunteers of both the sexes. However, the group of volunteers under investigation should be as homogeneous as possible to eliminate variability. According to USFDA Draft Guidance on Metformin (USFDA, 2012), healthy males and nonpregnant females from general population are recommended for the bioavailability study.

<u>Choice of Duration of Sampling</u>

The duration of sampling was chosen considering the Tmax and terminal elimination half-life of Metformin, which is 2.5 hours and 7.2 hours respectively (Meinicke, 2014). For Andrographolide, the half life was recorded at 1.5-2 hours. The sampling period corresponds to more than 4 of the longest reported half-lives for both the investigational products.

<u>Choice of Wash Out Period</u>

The minimum wash-out period of at least 7 days was also chosen according to the terminal elimination half-life of Metformin which is 7.2 hours. After more than 7-8 half-lives a pharmacokinetic carry-over effect can be excluded.

<u>Choice of Analytes</u>

Metformin and andrographolide will be measures in plasma and urine.

<u>Choice of Dosage</u>

Metformin will be conducted with 1000mg dose.

Andrographis paniculata will be conducted with 1000mg and 2000mg dose.

<u>Blinding</u>

This is an open label bioavailability study. Blinding procedure will not apply to this clinical trial. The investigator and the subjects aware on the type of investigational products administration during the clinical trials.

<u>Sample size calculation</u>

This is a phase 1 pharmacokinetics exploratory study to determine the rate and extent of absorption. A minimum of 12 subjects at each arm is sufficient to draw the pharmacokinetics profiles of the subjects. This is recommended by the USFDA guidance for bioavailability and bioequivalence study where minimum number of subjects are 12 volunteers. The study is using 3-period cross over design for 3 arms of investigational products.

4.3 Benefit-Risk Evaluation

The risk of this clinical trial are essentially the adverse effects for the investigational products. For Metformin tablet, subjects will be given the tablet together with 240ml of water. Andrographolide capsules will be given to the subjects according to the dose in the package insert recommendation that approved by Ministry of Healthy Malaysia. Subjects will be confined in CIC ward until 12 hours after post-dose to monitor any adverse events.

4.4 Selection of Study Population

A total of 18 healthy volunteers, 18 to 45 years of age (inclusive both), BMI between 18.5 – 29.5 kg/m^2 (inclusive both) with minimum weight of 50kg will be included in the study. Volunteers who fulfil all the inclusion criteria and exclusion criteria will be enrolled in the present study. Volunteers will be assigned enrolment numbers serially from 01 to 18 as per their reporting time (at check in after final selection) for period I which will remain the same throughout the study. This numbers will identify the volunteers and determine the sequence of drug administration as described in randomization schedule. To achieve 18 eligible subjects, approximately 25 or more subjects will be screened until 18 subjects eligible for the study.

4.4.1 Inclusion Criteria:

1. Sex: male
2. 18 to 45 years of age (inclusive both)
3. BMI 18.5 – 29.5 kg/m^2 (inclusive both) with minimum weight of 50kg
4. Non-smokers
5. Legible and willing to provide written informed consent.

4.4.2 Exclusion Criteria:

1. Volunteers suffering from any chronic illness such as arthritis, asthma, etc.
2. History of pre-existing bleeding disorder.
3. Clinically relevant abnormalities in the results of the laboratory screening evaluation.
4. Clinically significant abnormal ECG.
5. Positive HIV or positive hepatitis B or C in screening test or no known other hepatitis infection.
6. History of significant blood loss due to any reason, including blood donation in the past 3 months.
7. Participation in any study within past 3 months
8. History of alcohol or drug abuse
9. History of consumption of prescribed medication since last 14 days or OTC medication/ herbal remedies since last 7 days before beginning of the study.
10. Systolic blood pressure less than 100 mmHg or more than 139 mmHg and diastolic blood pressure less than 60 mmHg or more than 89 mmHg.
11. Pulse rate less than 60/minutes or more than 100/minute unless deem not clinically significant by investigator.
12. Oral temperature more than 37.5 degree Celsius.
13. History of allergy to the investigational product or any drug chemically similar to the drug under investigation.
14. Recent history of kidney or liver dysfunction.

15. Volunteers suffering from any psychiatric (acute or chronic) disorder.
16. Existence of any surgical or medical condition, which, in the judgment of the chief investigator and/or clinical investigator/physician, might interfere with the absorption, distribution, metabolism or excretion of the drug or likely to compromise the safety of volunteers.
17. Inability to communicate or co-operate.

4.4.3 Withdrawal Criteria

Volunteers will be informed that they are free to withdraw from the study at any time without giving any reason for doing so.

Volunteers may be discontinuing from the study for any of the following reasons:

- Volunteers not willing to continue with the study, irrespective of the reason.
- Volunteer experiences adverse event, when withdrawal would be in the best interest of the volunteer.
- Volunteer suffers from significant illness or undergo surgery during the study.
- Violation of the protocol by the volunteer.

Any volunteer may be discontinued from the study for any reason beneficial to his wellbeing.

The investigator will decide to discontinue any volunteer's participation in the study if, in his judgment, continuation in the study may prove harmful to the volunteer. Such a decision may be precipitated by adverse events, including changes in vital signs, physical examination and ECG. The investigator may also discontinue a volunteer due to poor compliance to the study protocol.

4.5 Study Medication

4.5.1 Identity of Investigational Products

Andrographis paniculata

Active ingredient	*Andrographis paniculata*
Brand	Shine Hempedu Bumi Capsule
Strength	500mg
Formulation	Capsule
Manufactured by	Herbal Science Sdn Bhd
Registration Number	MAL20002365T

Metformin

Name	Metformin
Brand	Glucophage
Strength	1000 mg

Formulation	Tablet
Manufactured by	MERCK Sdn. Bhd.
Registration number	MAL 06021194 AZ

4.5.2 Receipt, Storage and Retention

The study drug will be obtained from the manufacturer or retailer in sufficient quantities. Pharmacist shall document all the information about the receipt, storage, dispensing, administration, reconciliation, return and destruction of remaining study drugs and accountability of study drugs.

Details of study drugs used should include dosage form and strength, batch number/lot number, expiry date, and other coding that identifies the specific characteristics of the study drugs tested. Study drugs shall be stored under appropriate storage conditions as specified in the drug information.

All study drugs should be kept in CIC. Study drug shall be stored in wax-sealing bag containing the following label.

“FOR CLINICAL TRIAL PURPOSE ONLY”	
Protocol No: P1-PKPD-Metabolomics	Batch/Lot No:
Generic Name:	
Brand Name:	
Manufactured by:	
Storage Condition:	
Expiry date:	
Prepared by (Sign & Date)	

4.5.3 Method of Assigning Volunteers to Treatment Group

Volunteers who are eligible for the study will be first thoroughly informed about the objectives and details of the study. Each of the volunteer will be randomly assigned in a ratio of 1:1:1 into either *Andrographis paniculata* 1000mg, *Andrographis paniculata* 2000mg or Metformin 1000mg.

Dispensing:

The investigational products will be dispensed the day before the dosing in each period. The IP will be dispensed in presence of study personnel in pre-labelled sealing bag and will be delivered to study site before dosing by the Pharmacist. Extra 3 units of investigational product each shall be dispensed in each period.

Each label will contain study code, period (P), randomization code (RC), generic name of investigational product, batch/lot number, quantity (Qty), route of administration (ROA), enrolment no (En. No.), date of dosing (DOD), date of dispensing (DID), expiry date (Exp Date), manufactured by (Mfg By), storage condition and signature (sign) of pharmacist along with statement “FOR CLINICAL RESEARCH PURPOSE ONLY”. In the label of extra dispensing blank space shall be kept instead or enrolment number.

4.5.4 Randomization

The subjects will be randomised in the ratio of 1:1:1 into either *Andrographis paniculata* 1000mg capsules, *Andrographis paniculata* 2000mg capsules or Metformin 1000mg tablet. The order of receiving the investigational products for each subject during the periods of the study will be determined according to randomization schedule (Generated using computer software). The randomization will be performed in block and the code will be kept under controlled access. The personnel involved in dispensing of study drugs will be accountable for ensuring compliance to randomization schedule.

4.5.5 Drug Accountability

The investigator will not allow the study drugs to be used for purposes other than those specified in the protocol until the study ended. The drug accountability will be maintained by the pharmacist throughout the study.

4.5.6 Prior and Concomitant Medication

No concomitant drug therapy (including herbal remedies) will be allowed during the study except the drug used due to an adverse event. If any concomitant medicine is used then the investigator shall decide whether to withdraw or continue the volunteer based on the pharmacokinetic interaction of that drug with the study drug. Any intake of concomitant medication has to be documented in the adverse event form specifying the drug name and details.

5.0 STUDY PROCEDURES

5.1 Pre-Study Procedures

Demographic data, medical histories, physical examination, ECG, vital signs, hematology, biochemistry, urine analysis will be carried out for the screening. The screening evaluation will be valid for maximum of 3 months.

5.1.1 Demography Information

This will include age, sex, race, height, weight, BMI, consumption pattern of tea, caffeine, cola, alcohol, tobacco, grapefruit/ grapefruit juice, food habit etc. of the volunteers.

5.1.2 12-Leas Electrocardiogram

12 lead ECG will be done at the time of screening.

5.1.3 Medical Assessment of volunteers

This will include:

- Medical history and physical examination
- Significant past and family history

5.1.4 Vital Signs

At the time of screening oral temperature, pulse rate and blood pressure will be recorded.

5.1.5 Laboratory Tests

Laboratory tests will be done prior to the study during screening for all volunteers. These tests will involve hematology, biochemistry and urine analysis.

5.2 Planned Activity during the study

5.2.1 Enrolment

Following activities will be performed at the time of enrolment.

- Compliance check (for tobacco consumption, alcohol, grapefruit/grapefruit juice, beverage containing caffeine, xanthine or derivatives, OTC, herbal drugs or prescription medications etc.)
- Vital signs

Volunteers who will satisfy the inclusion and exclusion criteria will be enrolled in the study and provided with an enrolment number.

5.2.2 Housing

For each period, volunteers will be confined to the CIC for 12 hours post dose. The ward is equipped with patient beds, chairs, air-condition environment and recreational area.

5.2.3 Restrictions

<u>Medication</u>

If drug therapy other than that specified in the protocol is required prior to the study or during the study or in the washout period, decision shall be taken by the investigator whether to continue or discontinue the volunteer on the basis of the following:

- The pharmacology and pharmacokinetics of the non-study medication.
- The likelihood of drug-drug interaction, thereby affecting pharmacokinetic comparison of the study drugs.
- The time and duration of administration of the non-study medication and likelihood of interference in bio-analysis.
- The safety, well-being and clinical judgment about the volunteer.

Volunteers will be instructed not to take any prescribed medications beginning 14 days, OTC and herbal medications beginning 07 days, prior to initiation of study and until after the study completion, unless authorized by the chief investigator/clinical investigator.

<u>Diet and Water</u>

- Volunteers will be instructed to abstain from alcohol for 48 hours prior to dosing in period-1 until the end of last sample of last study period.
- Volunteers will be instructed to avoid beverages containing caffeine or xanthines (coffee, tea, cola drinks), food (containing chocolates) for 48 hours prior to dosing in period-1 until the end of last sample of the last study period.
- Volunteers will be instructed to avoid grapefruit/grapefruit juice for 7 days prior to dosing in period-! until the end of last sample of the last study period.

- Volunteers will be instructed to avoid tobacco in any form for 48 hours prior to dosing in period-1 until the end of last sample of last study period.
- Water will not be allowed for 1 hour before and up to 2 hours after study drug administration.
- At least 10 hours fasting will be required before dosing and 4 hours fasting will be required after dosing in each period.

Posture

Volunteers will be asked to remain in sitting/semi reclining position for at least first 4 hours after study drug administration except for blood sampling and toilet purpose whereby they would be allowed to walk for a brief period. However, if adverse events occur at any time volunteers may be placed in an appropriate position. The volunteers will be restricted from doing any sort of stressful physical activity during the entire period of stay at CIC ward.

5.2.4 Drug Administration

After an overnight fast of at least 10.0 hours, a single oral dose of test or reference product will be administered along with 240 mL of water at room temperature in each period based on randomization.

Dosing

Both the investigational products will be administered under the identical conditions in each period in presence of dosing supervisor. All volunteers will be instructed to swallow the study drugs without chewing. The actual time of drug administration will be documented. The time of dosing will be the time at which the investigational product is placed in the mouth of the volunteer.
All blood-sampling time points for each volunteer will be in accordance with the respective drug administration time.

Dosing Compliance
The person who is administering the study drug will check the compliance. To ensure the volunteer has swallowed the drug, mouth check will be done with torch. Additionally, glass check will be done. The labels will be prepared as flag labels. The personnel administering study drug will stick the detachable part of the label to the case record form with signature of that particular volunteer and person involved in the dosing.

5.2.5 Diet

During the CIC ward stay, food intake will be standardized and will be identical in both the periods. The first main meal (lunch) of the dosing day would be served not less than four hours after study drug administration. Lunch will be served at around 4.00 hours and the subsequent meals like snacks and dinner will be served at around 8 hours after drug administration according to the pre-defined food menu. Meals will be served individually, and the volunteers will be asked politely to finish the meal and the same will be recorded. Similar meals will be served in both the periods of the study to

all the volunteers. Dietary content and procedures during each study period will be identical.

5.2.6 Blood sampling for drug analysis

Blood Collection

A series of 15 venous blood samples will be collected over a 24.00 hour post dose period of time. To create a secure peripheral venous access, the indwelling intravenous cannula will be used for collection of pre-dose and up to 24 hours post-dose blood sample by syringe and transferred into tubes which contain KEDTA. Heparin-lock technique will be used to prevent clotting of the blood by injecting 0.5 mL-1mL of heparinized saline in the indwelling cannula. Before each blood sample is drawn from cannula, 0.5 mL-1mL of blood will be discarded to prevent the heparin from interfering with the analysis. If for any reason the indwelling cannula is blocked or must be removed for practical reasons, or on volunteer's request, direct vein-puncture will be done.

Blood collected in the polypropylene tubes will be shaken gently to ensure the proper mixing with anticoagulant. Sampling will relate to drug administration time.

In the morning of dosing day after recording vital signs, a pre-dose blood sample will be taken. Other blood samples will be withdrawn at following times: 0.5, 1, 1.5, 2, 2.5, 3, 3.5, 4, 5, 6, 8, 10, 12 and 24 hours post-dose (Ghozzi et al., 2015). Volunteers will return to give ambulatory sample at 24.00 hours post-dose. Ambulatory samples will be collected by direct vein-puncture. Blood sampling up to± 2 minutes of the planned time of in-house sampling and ±1 hour in ambulatory sample will be considered as an acceptable deviation. Beyond that, time deviation will be taken into consideration for further pharmacokinetic analysis, except for pre-dose samples, which will always be reported as zero-hour sample (0).

Total blood loss will be of approximately 500 mL during the entire study.

**Visit**	**Frequency**	**Volume (ml)**
blood for method validation	1	50
Each period blood sampling 15 x 10ml = 150ml	3	450
**Total**		500

In case, a blood sample collection time, vital signs time, RBS measurement, meal time, then the preferred order will be: vital signs, blood sampling, RBS measurement followed by meals.

Sample Handling Procedures

The blood samples will be collected in pre-labelled polypropylene tubes containing KEDTA for each mL of blood. Each sample will be centrifuged at 3500 RPM for 15 minutes at 20°C. The plasma then will be separated into two or three aliquots in cryotubes, which will be subsequently stored at approximately - 20°C/- 80°C freezer until it is analyzed.

Plasma samples will be stored at CIC ward during the study. After completion of the clinical phase of the study, Plasma samples will be transferred to bioanalytical laboratory by placing the vials in an insulated box containing coolant bags/dry ice.

Time of sample received, separation and storage will be documented.

All tubes containing blood samples & vials containing plasma samples will be labelled at least with the study code, enrolment no., period no., time point and date of dosing.

5.3 Adverse Events

5.3.1 Definitions

Adverse Event (AE): Any untoward medical occurrence in patient or clinical investigation subject administered pharmaceutical product and which does not necessarily have causal relationship with this treatment. An adverse event can therefore be any unfavorable and unintended sign (including abnormal laboratory finding), symptom or disease temporally associated with use of investigational product whether or not related to investigational product.

Abnormal laboratory values which are clinically significant (refer post-study evaluation) will also be reported as adverse event

Adverse Drug Reaction (ADR): All noxious and unintended responses to a medicinal product related to any dose should be considered adverse drug reactions.

Unexpected Adverse Drug Reaction: An adverse reaction, the nature or severity of which is not consistent with the applicable product information (e.g. Investigator's Brochure for an unapproved investigational product or package insert/summary of product characteristics for an approved product).

Expected Adverse Drug Reaction: An adverse reaction, nature or severity of which is consistent with applicable product information.

Serious Adverse Event (SAE): A serious adverse event (experience) or reaction is any untoward medical occurrence that at any dose:

- Results in death
- Is life-threatening
- Requires inpatient hospitalization (in case the study was being conducted on outpatient) or prolongation of existing hospitalization (in case the study was being conducted on in patient)
- Results in persistent or significant disability/incapacity, or
- In a congenital anomaly/birth defect

Study Related Injury or death: Any injury or death of the subject occurring in the study due to following reasons shall be considered as study related injury or death:

a) Adverse effect of investigational product(s);
b) Violation of approved protocol, scientific misconduct or negligence by the sponsor or his representative or the investigator;

c) Failure of investigational product to provide intended therapeutic effect; In case the standard care, though available, was not to be provided to the subject as per the clinical trial protocol;
d) Use of placebo in placebo-controlled trial; In case the standard care, though available, was not to be provided to the subject as per the clinical trial protocol;
e) Adverse effects due to concomitant medication excluding standard care, necessitated as part of approved protocol;
f) For injury to a child in-utero because of participation of parent in the study;
g) Any clinical procedures involved in the study.

5.3.2 Severity of adverse events

Severity of adverse events will be assessed as per the following classification:

Mild: Event results in mild or transient discomfort, not requiring intervention or treatment; does not limit or interfere with daily activities.

Moderate: Event is sufficiently discomforting so as to limit or interfere with daily activities; may require interventional treatment.

Severe: Event results in significant symptoms that prevents normal daily activities; may require hospitalization or invasive intervention.

5.3.3 Relationship to the Study Drugs

**Relationship**	**Description**
Unlikely	An Adverse event, including laboratory test abnormality, with a temporal relationship to drug administration which makes a causal relationship improbable, and in which other drugs, chemicals or underlying disease provide plausible explanations.
Possible	An Adverse event, including laboratory test abnormality, with a reasonable time sequence to administration of the drug, but which could also be explained by concurrent disease or other drugs or chemicals. Information on drug withdrawal (dechallenge) may be lacking or unclear.
Probable	An Adverse event, including laboratory test abnormality, with a reasonable time sequence to administration of the drug, unlikely to be attributed to concurrent disease or other drugs or chemicals, and which follows a clinically reasonable response on withdrawal (dechallenge). Rechallenge information is not required to fulfil this definition.
Definite	An Adverse event, including laboratory test abnormality, occurring in a reasonable time sequence to drug administration, and which cannot be explained by concurrent disease or other drugs or chemicals. The response to withdrawal of the drug (dechallenge) should be clinically plausible. The event must be definitive pharmacologically or phenomenologically, using a satisfactory rechallenge procedure if necessary.

Unassessable	An adverse event which cannot be judged because information is insufficient or contradictory, and which cannot be supplemented or verified.

5.3.4 Reporting and Documentation of Adverse Events

All adverse events including serious adverse events which occur within 14 days post discharge and considered to be related to the study drug must be reported and treated accordingly.

All SAE reporting will be done as per below mentioned procedure or as per applicable regulatory norms.

Reporting of serious adverse event: The investigator shall notify all serious adverse events (Telephonically, Fax, E-mail or written letter) to Ethics Committee within 24 hrs of its occurrence. The report of the serious adverse event after due analysis will be forwarded by the investigator to the chairman of the ethics committee within 14 days of occurrence of the serious adverse event.

Remaining all adverse events will be collected and recorded in the final report. Relationship/ causality used for reporting adverse events will be based on investigator's assessment of the event as unlikely, possible, probable, definite and assessable.

5.3.5 Emergency Procedures

Emergency equipment and medications will be available within the clinical unit. In case emergency treatment will be necessary, the treatment and the medications used during the emergency should be documented. To handle emergency, volunteer will be transferred with necessary precaution to emergency department at UMMC.

5.4 Post-study Procedures

5.4.1 Discharge from CIC ward

After completion of 24 hours after dosing, physical examination will be carried out to check the healthiness of volunteers. If they are found healthy, volunteers will be discharged from the facility. Volunteers withdrawn due to adverse event will be discharged based on physician’s discretion after physical examination.

5.4.2 Follow up

Any adverse event, which occurs in the course of the study, should be monitored and volunteers will be informed to come for follow-up according to the judgement of the investigator.

5.5 Volunteer Replacement

In case of withdrawn or dropout, volunteers will not be replaced. However, if in Period 1, a volunteer refuses his willingness to participate in the study after allotment of

enrolment number but before dosing, then another eligible volunteer will be enrolled in the study as per the in-house SOP and study specific requirements.

6.0 SAFETY ASSESSMENT

6.1 During Study Vital Sings

Vital signs (blood pressure and pulse rate) will be monitored at enrolment, pre-dose, 1.00, 12.00 and 24.00 hours post-dose, and whenever necessary in each study period. Additionally, oral temperature and respiratory rate will be monitored at enrolment, pre-dose and discharge of each period. Vital signs will be measured approximately 30 minus of the scheduled time except at pre-dose. For safety precautions random blood sugar (RBS) will be measured by glucometer at pre-dose as well as approximately at 1.00, 2.00, 3.00, 4.00, 24.00 hours post dose (with the last drop of respective time point blood sample collection) and whenever necessary. During the study, volunteers will be observed for any adverse event.

6.2 Physical Examination

At the time of check-in physical examination (along with medical history in period-1) and prior to discharge physical examination will be done. Fitness for dosing will be checked before each dosing. If unwell, the health status of the volunteers will be followed up until recovery; treatment will be given if necessary at the CIC ward.

6.3 Handling and Reporting of Adverse Event

Each volunteer will be asked for well-being at the time of vital signs monitoring at pre-dose, 1.00, 2.00, 4.00 and 24.00 hours post-dose. Volunteers shall be instructed to report any side effect (nature, severity, onset and disappearance) whenever it appears. In the case of an adverse event, investigator will decide whether to withdraw the volunteer from the study and to initiate appropriate treatment.

Management of Hypoglycemia:

20g of glucoses will be given to subjects at any point of time, in case of clinically significant hypoglycaemia and/ or symptoms as determined by the investigator.

7.0 ANALYSIS

The concentrations of Metformin and andrographolide will be quantified by means of validated LC MS/MS method.

The method should be evaluated for sufficient sensitivity and selectivity to determine concentration of Metformin and andrographolide. A limit of quantification should be set to quantify the levels of drug adequately in plasma. The method validation should include accuracy and precision evaluation with control samples at minimum four concentration levels. Further, stability of analyte also will be evaluated.

All samples of the same volunteer will be measured in a single analytical run to eliminate the influence of the inter-assay variance on the assessment. The final analytical report will be part of the final study report with concentration tables.

8.0 PHARMACOKINETIC EVALUATION

8.1 Pharmacokinetic Parameters

Individual listing of concentration data will be provided for all volunteers. Calculation of pharmacokinetic parameters, summary statistics and statistical analysis will be done with data of volunteers who complete at least one periods of the study.

The following pharmacokinetic parameters would be estimated using WinNonlin software

Cmax	Maximum concentration of drug observed in plasma
Tmax	Time required to reach maximum concentration of drug in plasma
AUC (0-t)	Area under the plasma concentration vs time curve from time zero to the last measurable concentration time t. It will be calculated using linear trapezoidal method.
AUC (0-inf)	Area under the plasma concentration vs time curve from time zero to time infinity.
T1/2	Time taken by plasma concentration to reduce to 50% during the elimination phase.

8.2 Treatment of Time Deviation

Blood sampling up to ± 2 minutes of the planned time of in-house sampling and ± 1 hour in ambulatory sample will be considered as an acceptable deviation. Beyond that, time deviation will be reported and taken into consideration for further pharmacokinetic analysis, except for pre-dose samples, which will always be reported as zero-hour sample (0).

9.0 STATISTICAL METHODS

9.1 Concentration-time profile and pharmacokinetic parameters

Plasma concentration vs. time data of andrographolide and Metformin will be provided.

Urine concentration vs. time data of andrographolide and Metformin will be provided.

The pharmacokinetic parameters Tmax, Cmax, AUC (0-t), AUC (0-inf), Kel and T1/2 will be calculated for andrographolide and Metformin.

In case of subjects where the terminal elimination phase is inadequately defined, pharmacokinetic parameters like AUC (0-inf), Kel and T1/2 will not be able to calculate.

9.2 Multivariate analysis

Various multivariate analysis will be perform to determine the secondary metabolites in the untargeted metabolomics analysis.

9.3 Handling of Dropouts or Missing Data

Missing sample can be due to withdrawal of volunteer, accidental spillage of samples or non-reporting of volunteer for ambulatory samples. The clinical data will clearly identify the missing samples. Missing values such as sample not submitted (SNS) or

sample not analysed (SNA) or non-reportable value (NRV) will be ignored (i.e., will not be interpolated by any method) from calculations of pharmacokinetics parameters.

9.4 Pre-dose Concentration

If the pre-dose concentration is ≤ 5% of Cmax value, the subject's data without any adjustment will be included in all pharmacokinetic measurements and calculations. If the pre-dose concentration is > 5% of Cmax value, the subject will be dropped from bioavailability study evaluations.

9.5 Data Due to Vomiting

Data from subjects who experience emesis at or before 2 times Tmax after dosing will be excluded from bioavailability analysis.

10.0 STUDY DOCUMENTATION

10.1 Final report

The study documentation will be kept in department of medicine and CIC ward by the student throughout the study.

On completion of the clinical, analytical and statistical phase of the study, the data will be used to prepare dissertation and final report.

10.2 Deviation from Protocol

All the deviations (an unintended departure from the study plan after study initiation) from approved protocol will be recorded and reported.

10.3 Archiving

All the study documents will be preserved until the study is completed and published.

11.0 ETHICAL AND LEGAL ASPECTS OF THE CONDUCT OF STUDY

11.1 Ethics

The investigators provide the University Malaya Medical Centre, Medical Ethics Committee (UMMC MEC) with all appropriate documents, including a copy of the protocol, informed consent form, patient information sheet, advertisement and any written information given to the subjects. The study will not begin until the investigator obtained the UMMC MEC written favourable approvals for the study.

If the protocol is amended, the changes must be approved by UMMC MEC prior to its implementation. If a revised Informed Consent Form is introduced during the study, the investigator shall obtain further consent from the subjects after UMMC MEC approved the ICF amendment.

11.2 Regulatory Requirements

The study will be conducted in accordance with Malaysian Guideline for Good Clinical Practice ((ICH), 2016) and comply to the Declaration of Helsinki (WMA, 2013). Both of the investigational medicine products are registered with Ministry of Health Malaysia.

This is a pharmacokinetic study that involved single dose oral administered of *Andrographis panaculata* on healthy volunteers, the used of *Andrographis paniculata* capsule in this clinical trial do not required CTIL and CTX.

The study also involved single dose oral administration of Metformin on healthy volunteers. This is an open label study, the product would not use or assembled in a way different from the approved form. Therefore, application of CTIL and CTX is not required for this study.

According to Malaysian Guideline for Clinical Trial Import Licence and Clinical Trial Exemption (CTIL/CTX), the following products will require a CTIL/CTX:

Section 3.2, A product with a marketing authorisation when used or assembled (formulated or packaged) in a way different from the approved form and when used for unapproved indication/ when use to gain further information about an approved use for clinical trial purpose.

section 3.3, a traditional product with a marketing authorisation with indication for “traditionally used” when used for unapproved indication/therapeutic claims for clinical trial purpose is required a CTIL/CTX.

12.0 PUBLICATION AND PRESENTATION

The study report shall form the basis of a manuscript for publication in a peer-reviewed journal. Authorship credit will be given to all investigators in the study program.

13.0 SUBJECT CONFIDENTIALITY

In order to maintain volunteer’s confidentiality, all data recorded during the course of the study will be identified by volunteer’s screening and enrolment number. Data relating to the study will be stored in such a way as to prevent their communication to the third party, except if required by regulatory and ethics committee without violating the confidentiality of the subjects, to the extent permitted by applicable laws and regulations. All data related to the project will be in the custody of the designated study personnel until transferred to archives.

14.0 MEDICAL CARE AND TREATMENT FOLLOWING STUDY COMPLETION

The study team will make a phone call to all the dosed subjects to ensure subject safety. The follow up safety information will be recorded into CRF.

15.0 APPENDICES

15.1 Appendix I: Declaration of Helsinki (WMA, 2013)

WMA DECLARATION OF HELSINKI – ETHICAL PRINCIPLES FOR MEDICAL RESEARCH INVOLVING HUMAN SUBJECTS

Adopted by the 18th WMA General Assembly, Helsinki, Finland, June 1964
and amended by the:
29th WMA General Assembly, Tokyo, Japan, October 1975
35th WMA General Assembly, Venice, Italy, October 1983
41st WMA General Assembly, Hong Kong, September 1989
48th WMA General Assembly, Somerset West, Republic of South Africa, October 1996
52nd WMA General Assembly, Edinburgh, Scotland, October 2000
53rd WMA General Assembly, Washington DC, USA, October 2002 (Note of Clarification added)
55th WMA General Assembly, Tokyo, Japan, October 2004 (Note of Clarification added)
59th WMA General Assembly, Seoul, Republic of Korea, October 2008
64th WMA General Assembly, Fortaleza, Brazil, October 2013

Preamble

1. The World Medical Association (WMA) has developed the Declaration of Helsinki as a statement of ethical principles for medical research involving human subjects, including research on identifiable human material and data.

The Declaration is intended to be read as a whole and each of its constituent paragraphs should be applied with consideration of all other relevant paragraphs.

2. Consistent with the mandate of the WMA, the Declaration is addressed primarily to physicians. The WMA encourages others who are involved in medical research involving human subjects to adopt these principles.

General Principles

3. The Declaration of Geneva of the WMA binds the physician with the words, “The health of my patient will be my first consideration,” and the International Code of Medical Ethics declares that, “A physician shall act in the patient’s best interest when providing medical care.”

4. It is the duty of the physician to promote and safeguard the health, well-being and rights of patients, including those who are involved in medical research. The physician’s knowledge and conscience are dedicated to the fulfilment of this duty.

5. Medical progress is based on research that ultimately must include studies involving human subjects.

6. The primary purpose of medical research involving human subjects is to understand the causes, development and effects of diseases and improve preventive, diagnostic and therapeutic interventions (methods, procedures and treatments). Even the best proven interventions must be evaluated continually through research for their safety, effectiveness, efficiency, accessibility and quality.

7. Medical research is subject to ethical standards that promote and ensure respect for all human subjects and protect their health and rights.

8. While the primary purpose of medical research is to generate new knowledge, this goal can never take precedence over the rights and interests of individual research subjects.

9. It is the duty of physicians who are involved in medical research to protect the life, health, dignity, integrity, right to self-determination, privacy, and confidentiality of personal information of research subjects. The responsibility for the protection of research subjects must always rest with the physician or other health care professionals and never with the research subjects, even though they have given consent.

10. Physicians must consider the ethical, legal and regulatory norms and standards for research involving human subjects in their own countries as well as applicable international norms and standards. No national or international ethical, legal or regulatory requirement should reduce or eliminate any of the protections for research subjects set forth in this Declaration.

11. Medical research should be conducted in a manner that minimises possible harm to the environment.

12. Medical research involving human subjects must be conducted only by individuals with the appropriate ethics and scientific education, training and qualifications. Research on patients or healthy volunteers requires the supervision of a competent and appropriately qualified physician or other health care professional.

13. Groups that are underrepresented in medical research should be provided appropriate access to participation in research.

14. Physicians who combine medical research with medical care should involve their patients in research only to the extent that this is justified by its potential preventive, diagnostic or therapeutic value and if the physician has good reason to believe that participation in the research study will not adversely affect the health of the patients who serve as research subjects.

15. Appropriate compensation and treatment for subjects who are harmed as a result of participating in research must be ensured.

Risks, Burdens and Benefits

16. In medical practice and in medical research, most interventions involve risks and burdens.

Medical research involving human subjects may only be conducted if the importance of the objective outweighs the risks and burdens to the research subjects.

17. All medical research involving human subjects must be preceded by careful assessment of predictable risks and burdens to the individuals and groups involved in the research in comparison with foreseeable benefits to them and to other individuals or groups affected by the condition under investigation.

Measures to minimise the risks must be implemented. The risks must be continuously monitored, assessed and documented by the researcher.

18. Physicians may not be involved in a research study involving human subjects unless they are confident that the risks have been adequately assessed and can be satisfactorily managed.

When the risks are found to outweigh the potential benefits or when there is conclusive proof of definitive outcomes, physicians must assess whether to continue, modify or immediately stop the study.

Vulnerable Groups and Individuals

19. Some groups and individuals are particularly vulnerable and may have an increased likelihood of being wronged or of incurring additional harm.

All vulnerable groups and individuals should receive specifically considered protection.

20. Medical research with a vulnerable group is only justified if the research is responsive to the health needs or priorities of this group and the research cannot be carried out in a non-vulnerable group. In addition, this group should stand to benefit from the knowledge, practices or interventions that result from the research.

Scientific Requirements and Research Protocols

21. Medical research involving human subjects must conform to generally accepted scientific principles, be based on a thorough knowledge of the scientific literature, other relevant sources of information, and adequate laboratory and, as appropriate, animal experimentation. The welfare of animals used for research must be respected.

22. The design and performance of each research study involving human subjects must be clearly described and justified in a research protocol.

The protocol should contain a statement of the ethical considerations involved and should indicate how the principles in this Declaration have been addressed. The protocol should include information regarding funding, sponsors, institutional affiliations, potential conflicts of interest, incentives for subjects and information regarding provisions for treating and/or compensating subjects who are harmed as a consequence of participation in the research study.

In clinical trials, the protocol must also describe appropriate arrangements for post-trial provisions.

Research Ethics Committees

23. The research protocol must be submitted for consideration, comment, guidance and approval to the concerned research ethics committee before the study begins. This committee must be transparent in its functioning, must be independent of the researcher, the sponsor and any other undue influence and must be duly qualified. It must take into consideration the laws and regulations of the country or countries in which the research is to be performed as well as applicable international norms and standards but these must not be allowed to reduce or eliminate any of the protections for research subjects set forth in this Declaration.

The committee must have the right to monitor ongoing studies. The researcher must provide monitoring information to the committee, especially information about any serious adverse events. No amendment to the protocol may be made without consideration and approval by the committee. After the end of the study, the researchers must submit a final report to the committee containing a summary of the study’s findings and conclusions.

Privacy and Confidentiality

24. Every precaution must be taken to protect the privacy of research subjects and the confidentiality of their personal information.

Informed Consent

25. Participation by individuals capable of giving informed consent as subjects in medical research must be voluntary. Although it may be appropriate to consult family members or community leaders, no individual capable of giving informed consent may be enrolled in a research study unless he or she freely agrees.

26. In medical research involving human subjects capable of giving informed consent, each potential subject must be adequately informed of the aims, methods, sources of funding, any possible conflicts of interest, institutional affiliations of the researcher, the anticipated benefits and potential risks of the study and the discomfort it may entail, post-study provisions and any other relevant aspects of the study. The potential subject must be informed of the right to refuse to participate in the study or to withdraw consent to participate at any time without reprisal. Special attention should

be given to the specific information needs of individual potential subjects as well as to the methods used to deliver the information.

After ensuring that the potential subject has understood the information, the physician or another appropriately qualified individual must then seek the potential subject's freely-given informed consent, preferably in writing. If the consent cannot be expressed in writing, the non-written consent must be formally documented and witnessed.

All medical research subjects should be given the option of being informed about the general outcome and results of the study.

27. When seeking informed consent for participation in a research study the physician must be particularly cautious if the potential subject is in a dependent relationship with the physician or may consent under duress. In such situations the informed consent must be sought by an appropriately qualified individual who is completely independent of this relationship.

28. For a potential research subject who is incapable of giving informed consent, the physician must seek informed consent from the legally authorised representative. These individuals must not be included in a research study that has no likelihood of benefit for them unless it is intended to promote the health of the group represented by the potential subject, the research cannot instead be performed with persons capable of providing informed consent, and the research entails only minimal risk and minimal burden.

29. When a potential research subject who is deemed incapable of giving informed consent is able to give assent to decisions about participation in research, the physician must seek that assent in addition to the consent of the legally authorised representative. The potential subject's dissent should be respected.

30. Research involving subjects who are physically or mentally incapable of giving consent, for example, unconscious patients, may be done only if the physical or mental condition that prevents giving informed consent is a necessary characteristic of the research group. In such circumstances the physician must seek informed consent from the legally authorised representative. If no such representative is available and if the research cannot be delayed, the study may proceed without informed consent provided that the specific reasons for involving subjects with a condition that renders them unable to give informed consent have been stated in the research protocol and the study has been approved by a research ethics committee. Consent to remain in the research must be obtained as soon as possible from the subject or a legally authorised representative.

31. The physician must fully inform the patient which aspects of their care are related to the research. The refusal of a patient to participate in a study or the patient's

decision to withdraw from the study must never adversely affect the patient-physician relationship.

32. For medical research using identifiable human material or data, such as research on material or data contained in biobanks or similar repositories, physicians must seek informed consent for its collection, storage and/or reuse. There may be exceptional situations where consent would be impossible or impracticable to obtain for such research. In such situations the research may be done only after consideration and approval of a research ethics committee.

Use of Placebo

33. The benefits, risks, burdens and effectiveness of a new intervention must be tested against those of the best proven intervention(s), except in the following circumstances:

Where no proven intervention exists, the use of placebo, or no intervention, is acceptable; or

Where for compelling and scientifically sound methodological reasons the use of any intervention less effective than the best proven one, the use of placebo, or no intervention is necessary to determine the efficacy or safety of an intervention

and the patients who receive any intervention less effective than the best proven one, placebo, or no intervention will not be subject to additional risks of serious or irreversible harm as a result of not receiving the best proven intervention.

Extreme care must be taken to avoid abuse of this option.

Post-Trial Provisions

34. In advance of a clinical trial, sponsors, researchers and host country governments should make provisions for post-trial access for all participants who still need an intervention identified as beneficial in the trial. This information must also be disclosed to participants during the informed consent process.

Research Registration and Publication and Dissemination of Results

35. Every research study involving human subjects must be registered in a publicly accessible database before recruitment of the first subject.

36. Researchers, authors, sponsors, editors and publishers all have ethical obligations with regard to the publication and dissemination of the results of research. Researchers have a duty to make publicly available the results of their research on human subjects and are accountable for the completeness and accuracy of their reports. All parties should adhere to accepted guidelines for ethical reporting. Negative and inconclusive as well as positive results must be published or otherwise made publicly available. Sources of funding, institutional affiliations and conflicts of interest

must be declared in the publication. Reports of research not in accordance with the principles of this Declaration should not be accepted for publication.

Unproven Interventions in Clinical Practice

37. In the treatment of an individual patient, where proven interventions do not exist or other known interventions have been ineffective, the physician, after seeking expert advice, with informed consent from the patient or a legally authorised representative, may use an unproven intervention if in the physician's judgement it offers hope of saving life, re-establishing health or alleviating suffering. This intervention should subsequently be made the object of research, designed to evaluate its safety and efficacy. In all cases, new information must be recorded and, where appropriate, made publicly available.

16.0 REFERENCES:

(ICH), I. C. O. H. 2016. Integrated Addendum to ICH E6(R1): Guideline for Good Clinical Practice E6(R2).

AGARWAL, R., SULAIMAN, S. A. & MOHAMED, M. 2005. Open label clinical trial to study adverse effects and tolerance to dry powder of the aerial part of andrographis paniculata in patients type 2 with diabetes mellitus. *The Malaysian journal of medical sciences : MJMS,* 12**,** 13-19.

FOO, M. E., ANBU, P., GOPINATH, S. C. B., LAKSHMIPRIYA, T., LEE, C.-G., YUN, H. S., UDA, M. N. A. & YAAKUB, A. R. W. 2018. Antimicrobial activity of functionalized single-walled carbon nanotube with herbal extract of Hempedu bumi. *Surface and Interface Analysis,* 50**,** 354-361.

GHOZZI, H., HAMMAMI, S., AFFES, H., KSOUDA, K., SAHNOUN, Z., HAKIM, A. & ABID, M. 2015. Bioequivalence evaluation of glibenclamide 5-mg tablets: diabenil(R) and daonil(R) (in 24 healthy volunteers). *Tunis Med,* 93**,** 96-100.

HAO, D. C., GE, G. B., XIAO, P. G., WANG, P. & YANG, L. 2015. Drug metabolism and pharmacokinetic diversity of ranunculaceae medicinal compounds. *Curr Drug Metab,* 16**,** 294-321.

KANTAE, V., KREKELS, E. H. J., ESDONK, M. J. V., LINDENBURG, P., HARMS, A. C., KNIBBE, C. A. J., VAN DER GRAAF, P. H. & HANKEMEIER, T. 2017. Integration of pharmacometabolomics with pharmacokinetics and pharmacodynamics: towards personalized drug therapy. *Metabolomics,* 13**,** 9.

MEINICKE, T. 2014. Bioequivalence of Glucophage® (Metformin) Tablets from Europe and the United States Tested in Healthy Volunteers. *Journal of Bioequivalence & Bioavailability,* 06.

MELCHIOR, J., SPASOV, A. A., OSTROVSKIJ, O. V., BULANOV, A. E. & WIKMAN, G. 2000. Double-blind, placebo-controlled pilot and phase III study of activity of standardized Andrographis paniculata Herba Nees extract fixed combination (Kan jang) in the treatment of uncomplicated upper-respiratory tract infection. *Phytomedicine,* 7**,** 341-50.

MIAO, Z. X. & WANG, Y. H. 2013. [Application of NMR technique in the discovery and pharmacological studies of active substances from natural products]. *Yao Xue Xue Bao,* 48**,** 1383-9.

NAJIB NIK, A. R. N., FURUTA, T., KOJIMA, S., TAKANE, K. & ALI MOHD, M. 1999. Antimalarial activity of extracts of Malaysian medicinal plants. *J Ethnopharmacol,* 64**,** 249-54.

SANDBORN, W. J., TARGAN, S. R., BYERS, V. S., RUTTY, D. A., MU, H., ZHANG, X. & TANG, T. 2013. Andrographis paniculata extract (HMPL-004) for active ulcerative colitis. *Am J Gastroenterol,* 108**,** 90-8.

SHERIDAN, H., KRENN, L., JIANG, R., SUTHERLAND, I., IGNATOVA, S., MARMANN, A., LIANG, X. & SENDKER, J. 2012. The potential of metabolic fingerprinting as a tool for the modernisation of TCM preparations. *J Ethnopharmacol,* 140**,** 482-91.

USFDA 2012. Draft Guidance on Glyburide. Mac 2012 ed.: USFDA.

WMA. 2013. *WMA Declaration of Helsinki - Ethical Principles for Medical Research Involving Human Subjects* [Online]. World Medical Association, Inc. Available: http://www.wma.net/en/30publications/10policies/b3/ [Accessed July 5, 2013].

WU, H., LI, X., YAN, X., AN, L., LUO, K., SHAO, M., JIANG, Y., XIE, R. & FENG, F. 2015. An untargeted metabolomics-driven approach based on LC-TOF/MS and LC-MS/MS for the screening of xenobiotics and metabolites of Zhi-Zi-Da-Huang decoction in rat plasma. *J Pharm Biomed Anal,* 115**,** 315-22.
