# Supplementary material for: Pharmacokinetics and Metabolomic Profiling of Metformin and Andrographis paniculata: A Protocol for a Crossover Randomised Controlled Trial
Source: J Clin Med. 2022 Jul 6;11(14):3931. doi: 10.3390/jcm11143931 (PMC9323336; doi:10.3390/jcm11143931)
Supplement: Supplementary file 1 [file jcm-11-03931-s001.zip › Document S2. P1-PKPD-Metabolomic ICF-English V2.0 160719.pdf]

Subject Consent Form

I, ……………………………………………………………………………….. have read and understood the above Subject Information Sheet, and hereby voluntarily consent to participate in this research study "A Phase 1, Open Label, Randomized, Three-Period, Crossover, Single Dose Oral Administration Of *Andrographis Paniculata* And Metformin Clinical Trial In Healthy Volunteers Under Fasting Condition.".

1. I am participating in this study on my own free will, and I understand the nature, aim, as well as the possible side effects that may arise during this study.
2. Detailed explanation as to how this research will be carried out has been given to me and also has been explained to me orally. This includes the duration of the study, the tests and procedures that will be performed, as well as any discomfort that may arise.
3. I understand that even though urine pregnancy test will be conducted before the study (if applicable), the test will not be able to detect early pregnancy.
4. I know that I can ask questions regarding the study at any time before, during and after the study.
5. I have been told that no information regarding the history of my illness will be revealed to the public, and that the results of the completed study will be published in such a way as to keep my identity unknown.
6. I understand that I am free to withdraw from this study at any stage.
7. By signing the subject consent form, I indicate that I have read and understood the information about the use of remaining plasma for general research.
8. Finally, I hereby acknowledge that I have received a copy of this consent form after signing it.

Name of participant:	Signature:
NRIC No:	Date:

Name of investigator/designee:	Signature:
NRIC No:	Date:

I declare that the information in the consent form and any other written information has been accurately explained to, and appropriately understood by the subject, and the informed consent was freely given to the subject.

Name of investigator/designee:	Signature:
NRIC No:	Date:
