# Supplementary material for: Pharmacokinetics and Metabolomic Profiling of Metformin and Andrographis paniculata: A Protocol for a Crossover Randomised Controlled Trial
Source: J Clin Med. 2022 Jul 6;11(14):3931. doi: 10.3390/jcm11143931 (PMC9323336; doi:10.3390/jcm11143931)
Supplement: Supplementary file 1 [file jcm-11-03931-s001.zip › Document S3. P1-PKPD-Metabolomic PIS-English V2.0 160719.pdf]

# INFORMATION SHEET TO TRIAL SUBJECTS

| | |
|---|---|
| NAME OF STUDY: | A Phase 1, Open Label, Randomized, Three-Period, Crossover, Single Dose Oral Administration Of *Andrographis Paniculata* And Metformin Clinical Trial In Healthy Volunteers Under Fasting Condition. |
| STUDY NUMBER: | P1-PKPD-Metabolomic |
| STUDY DOCTOR (INVESTIGATOR): | Dr. Luqman bin Ibrahim<br>University Malaya Medical Centre<br>Lembah Pantai,<br>59100 Kuala Lumpur.<br>Tel: +6012-3812977<br>Email: luqmanibrahim@luqmanibrahim.com<br><br>Prof. Madya Dr. Hasniza Zaman Huri<br>Clinical Investigation Center (CIC),<br>University Malaya Medical Centre<br>Lembah Pantai,<br>59100 Kuala Lumpur.<br>Office Hours Tel: +603-7949 4932<br>Email: hasnizazh@um.edu.my<br><br>Tee Khim Boon<br>University Malaya Medical Centre<br>Lembah Pantai,<br>59100 Kuala Lumpur.<br>Tel: +6012-6930692 (Ms Tee)<br>Email: mva180021@siswa.um.edu.my |
| ETHICS COMMITTEE (EC): | University Malaya Medical Centre, Medical Research Ethics Committee (UMMC MREC)<br>Lembah Pantai,<br>59100 Kuala Lumpur.<br>Office Hours Tel: +603-79492251/3209 |

| | |
|---|---|
| **Protocol Number:** | **P1-PKPD-Metabolomics** |
| **Version:** | **2.0** |
| **Date:** | **16 July 2019** |

## Invitation:

You are being asked to consider whether you would like to participate in a clinical research. A clinical research is an experimental study of a new therapy, procedure, or drug and only includes people who want to be in the study. The following information describes the study and your role as a possible participant. Please read this information carefully and do not hesitate to ask your study doctor any questions to ensure that you are able to make an informed decision as to whether to participate.

You are under no obligation to take part in this study and should you decide not to, your future treatment will not be affected in any way.

**What is the purpose of this clinical research?**

The purpose of this clinical trial is to measure the rate and extend of compounds absorbed into body after single dose oral administration of *Andrographis paniculata* or Metformin. This study is to identify the pharmacokinetics and pharmacodynamics profile of *Andrographis paniculata* (King of Bitters) and Metformin (Diabetes Medicine).

The study products do not contain porcine, bovine or animal components.

**How many people will be in the study?**

This study will screen for approximately 25 healthy volunteers for eligibility in and exclusion criteria. It was expected that approximately 18 healthy volunteers would be enrolled in this study at Clinical Investigation Centre, University Malaya Medical Centre.

**What will happen to me if I take part?**

If you agree and give consent to take part in this study, your suitability for participation in the study will be checked (called screening) before receiving any study drug. Your medical records will be reviewed to confirm your suitability and applicable tests and procedures will be conducted.

**During the study**

If you are suitable to take part in this study and receive study medication, you will be asked to make approximately 3 study periods to the study site over the next 3 weeks. You may be asked to make additional visit for study procedure at your study doctor's discretion for safety follow up. Each study period will require you to stay over from 7.00am to 8.00pm and ambulatory single blood taking at 8am on the following day. This study should take approximately 4 weeks to be completed.

**Study period**

You will be admitted into the CIC Ward on 6:30-7:00 am. The ward is equipped with patient beds, chairs, air-condition environment and recreational area. You will need to report to the investigators any medical condition you may have experienced since your last visit to the site. Your body temperature and vital signs (blood pressure, heart rate and respiratory rate) will be checked.

On the first day, a small tube will be inserted into a suitable vein preferably on your arm for collection of blood. After which you will be randomly given 1 tablet/capsule of either the *Andrographis panaculata* or Metformin to be taken by mouth with a glass of water. The randomisation is like the toss of a coin and it is generated by a computer

program. Following that, you will spend a whole day in the study ward in order to allow the investigation team to obtain 10ml (approximately 2 tea spoonsful) of blood from you at specified times, e.g. within 60 minutes before dosing, and at 0.5, 1, 1.5, 2, 2.5, 3, 3.5, 4, 5, 6, 8, 10, 12 and 24 hours following drug administration. After each blood collection, about 1-2 ml of heparinised saline (a salt solution containing a small amount of drug that reduces blood clotting) will be injected into your bloodstream through the small tube that has been inserted to prevent blood clots at the small tube. All blood samples will be taken while you are in the study ward. The total volume of blood that will be collected each period is 150 ml (around 10 tablespoon) and the total volume of blood that will be collected for the entire study (including the 10 ml of blood drawn for medical screening) is 500 ml (around 34 tablespoonfuls).

| **Visit** | **Frequency** | **Volume (ml)** |
|---|---|---|
| Method validation | 1 | 50 |
| During dosing 15x10mL | 3 | 450 |
| **Total** | | **500 ml** |

Throughout the study period, your vital signs will be checked at 3 times following drug administration. You are required to report to the investigators any discomfort that happens to you during the study.

You will be discharged after the 12 hours blood collection. You are required to come to CIC at 24 post dose for ambulatory blood collection. After at least 1 week break to allow your body to sufficiently remove the dr ug that you have taken, you will be admitted into the CIC ward again and you will be switched over to take the other product (crossover) in the same manner.

You are also will receive a phone call or visit to ward CIC approximately a week after last blood draw in the final study period for a safety follow-up if you have taken the study drug, regardless of whether or not you are discontinued early. During the safety monitoring, you will be interviewed and examined briefly. You are reminded to report to the investigators any discomfort that happens to you since being discharged from the study.

The blood sample obtained from you will be separated into different parts. The plasma will be used for the study and the blood cells will be treated as clinical waste which will be discarded. The remaining plasma will be pooled and used for general research and development purpose such as method development of a chemical compound (i.e. to check and prove the accuracy of detecting a specific chemical compound in the plasma). Your identity will not be traceable.

## Restrictions

Foods and drinks: You will have to follow a standard diet during your stay in the study ward. No food or drink except plain water will be allowed from at least 8 hours before dosing until 4 hours after dosing. Meals will be identical for all study periods.

Except for the glass of water given with study drug, no drink will be allowed from 1 hour before dosing until 1 hour after dosing. Water will be provided at one’s pleasure at all other times.

Physical activity: You will need to maintain an upright position (standing or sitting) for the first 20 minutes after taking the study drug. You will have to avoid vigorous physical activity at all times during your stay in the study ward.

Sexual activity: Female subjects of childbearing potential must use acceptable method of birth control from 14 days prior to dosing for the first study period until after the safety follow-up monitoring. Acceptable methods of birth control:

- Double barrier (one by each partner) and at least one of these barriers must contain spermicide (substance that kills sperm):
  - The male must use a condom
  - The female may select one of the following: cervical cap, diaphragm or sponge (a female condom is not an option because of the risk of tearing when both partners use a condom)
- Hormonal (oral pill, injection, patch, vaginal ring or implant)
- Intrauterine contraceptive system (small device that is inserted into the uterus/womb)
- Surgical (vasectomy for men or tubal ligation for women)
- Refrain from sexual activity

Other restrictions:

You will need to abstain from:

- Taking alcohol-based products from 24 hours prior to dosing until after the last sample collection of each study period;
- Taking food or beverage containing xanthine derivatives or xanthine-related compounds such as coffee, tea, chocolate, cola, Milo or energy drink from 24 hours prior to dosing until after the last sample collection of each study period;
- Taking food or beverage containing grapefruit (including marmalade) or pomelo from 7 days prior to dosing until after the last sample collection of the study;
- Smoking cigarettes or taking nicotine products from 24 hours prior to dosing until after the last sample collection of each study period;
- Taking prescription drugs and over-the-counter (OTC) products (including herbal remedies) from 7 days prior to dosing for the 1st study period until after the safety follow-up, except for birth control medications.

It is important that you respect all restrictions since that may affect the study results or may compromise your safety. All deviations to these restrictions should be reported as soon as possible to the investigators. If you have food restrictions or allergies, you may not participate in this research study.

Upon each admission to CIC ward, a staff member will check your personal belongings to make sure you do not bring any forbidden item (e.g. cigarettes, medications, foods and drinks) into the study ward.

**What will I have to do?**

It is important that you answer all the questions asked by the study staff honestly and completely. It is important that you inform your study doctor of any changes to your health, whether you think it is related to the study drug. Also, any changes to your medication, either prescribed by your family doctor or those you buy at a pharmacy, including herbal or alternative therapies or remedies, should be mentioned to your study doctor. You should ask your study doctor before taking any new medications and always comply with your study doctor's instructions during the clinical trial. For your safety, it is important that you keep all appointments at the study site. You should call the study staff if you have any questions or concerns.

You will be given a healthy volunteer card by your study doctor which you should carry with you during the clinical trial. This will provide details of the study and the contact number of your study doctor.

**What is the drug that is being tested?**

Two types of products are being tested. The products are Metformin and *Andrographis paniculata*

Metformin

Metformin has been approved and marketed in Malaysia. Metformin is used to control blood glucose levels in patients with type 2 diabetes mellitus.

*Andrographis panicula*ta (King of Bitter)

*Andrographis panicula* also known as hempedu bumi or King of bitter. It is a traditional product that approved by Ministry of Healthy Malaysia and marketed in Malaysia. The product was traditionally used to relief fever, cough, flu and sore throat.

**What are the potential risks and discomforts?**

Metformin

Metformin has presented a good safety profile and is usually well tolerated. Some common adverse reactions associated with Metformin include

- Digestive problems, such as feeling sick (nausea), being sick (vomiting), diarrhoea, bellyache (abdominal pain) and loss of appetite.
- Lactic acidosis. Symptoms of lactic acidosis are vomiting, bellyache (abdominal pain) with muscle cramps, a general feeling of not being well with severe tiredness, and difficulty in breathing.

- Abnormalities in liver function tests or hepatitis (inflammation of the liver; this may cause tiredness, loss of appetite, weight loss, with or without yellowing of the skin or whites of the eyes).
- Skin reactions such as redness of the skin (erythema), itching or an itchy rash (hives).
- Low vitamin B12 levels in the blood.

*Andrographis panicula* (Hempedu bumi)

King of bitter is a herbal product that are general safe to be consumed. You can purchase the herbal product from pharmacy for traditionally use. The common side effects are as below:

- Orally administration in high dose might result in gastric discomfort, vomiting and loss of appetite. These side effects appear to be due to the bitter taste of andrographolide.
- Two cases of urticaria have been reported.

**What are the advantages and disadvantages of participation in the study?**

No clinical or therapeutic benefit should be expected of the study drug taken. However, by taking part, you will provide new information that may benefit the treatment of other patients with this drug. You will be given close attention from the study staff during the time you are involved in the study. You may receive information about your health from physical examinations and medical tests done in this study.

**Who can I contact with further questions?**

You may freely ask questions about this consent form or the study at any time. If you have additional questions, or experience a research-related injury, contact the study doctor using the details provided on the first page of this information sheet.

If you have a complaint or question about your rights as a research subject, you may contact the University Malaya Medical Centre, Medical Research Ethics Committee (UMMC MREC) using the details provided on the first page of this information sheet. This is a group of scientific and non-scientific individuals who reviewed research studies with the safety and welfare of research subjects in mind and gave their positive opinion to this research.

**What happens if I change my mind?**

Your participation in this study is voluntary. You do not have to take part, or you may discontinue your involvement at any time without penalty or loss of benefits to which you are otherwise entitled. If you decide to leave the study, tell the study doctor and follow instructions. It may be helpful if you could explain your reasons but you do not

have to tell anyone why you do not want to be in the study, However, if you leave the study because of an adverse event (any discomfort or decline of your health), it is very important that you tell the study doctor about the adverse event. Your decision not to participate or to leave the study will not affect the care you receive now or in the future. You may receive standard treatment and no prejudice will be shown towards you for medical care or participation in future research.

In addition, your study doctor may withdraw you from the study for your own safety, even if you wish to continue to participate, for example:

- If you need additional medication
- If you experience a study related injury
- If you are unwilling or unable to follow the rules of the study (e.g., not keeping study appointment)
- If you are female and become pregnant
- At the request of ethics committee or the regulatory authority
- If you withdraw consent
- If you have any adverse event that would compromise your safety if you continue to participate in the study
- If you do not follow the study protocol

If this happens, your study doctor will explain the reasons.

If your participation in the study is stopped early, you may be asked to complete end of study procedures (such as safety follow up call) for your own safety.

**Are there any costs if I decide to participate?**

You will not receive payment for participating in this study, but you will not be required to pay for any study procedures and laboratory testing. Food during your stay in the study ward will be provided at no cost to you. You will be paid per study visit to compensate you for your time, blood loss and for any travel expenses.

| | |
|---|---|
| Screening visit | RM 50 |
| Blood for method validation | RM 50 |
| Study Visit 1 | RM 55 |
| 15 plasma samples x RM 5 | RM 75 |
| 4 urine samples x RM 5 | RM 20 |
| Study Visit 2 | RM 55 |
| 15 plasma samples x RM 5 | RM 75 |
| 4 urine samples x RM 5 | RM 20 |
| Study Visit 3 | RM 55 |
| 15 plasma samples x RM 5 | RM 75 |
| 4 urine samples x RM 5 | RM 20 |
| Completed whole study | RM 100 |
| **Total** | **RM 650** |
| Safety follow (If Required) | RM 50 |

You will be paid RM 650 if you complete this study and additional RM 50 if safety follow visit is required. You will be paid proportionately if you do not complete the whole study or if you fail to abide by the study schedule. Your reimbursement will be distributed in proportionate payment according to the table above.

**RESERVE SUBJECT**

You will be informed if you are being enrolled into this study as a reserve subject. Reserve subjects are additional subjects who may be enrolled into the study as standbys to replace subjects who drop out before the first dosing. Research subjects will be admitted into the study ward on Day 1 of the first study period and undergo all study procedures like other confirmed subjects. If there is subject dropout before the first dosing, a reserve subject will take over the place of the subject and continue the entire study procedures. If there is no subject dropout, the reserve subjects will them be discharged accordingly after all the subjects have been dosed and they will not be required to return for the next study period and safety follow-up. A reserve subject will be compensated RM 50 for the inconvenience caused.

**How will my confidentiality be respected and the privacy of my personal information maintained?**

The study site will record basic details about you, including your name, contact details, gender, height, weight and racial origin (only for clinical research purpose), as well as information on your medical history, and clinical data collected about your participation in the study. The following people may also access these records:

- Ethical committee that approved this study and ensures that your rights and well-being are safeguarded.
- Regulatory authority involved in keeping research safe for participants.
- All personnel accessing your records are required to respect your confidentiality at all times.

Under the Laws of Malaysia, Act 709, Personal Data Protection Act 2010, you may request for access to or correction of your personal data or limit the processing of the same at any time hereafter by submitting such request to your study doctor. Any inquiries or complaints with respect to your personal data should be channelled to your study doctor in this manner.

Please note that it is necessary for us to process your personal data, without which you may not be able to continue participating in this research study. You agree in that the terms herein shall supersede any prior consents or agreements in relation to the processing of your personal data in relation to this research study and other related purposes.

If you should withdraw from the study, data collected prior to your withdrawal may still be processed along with data collected as part of the study. Normally no new information will be collected for the study database unless you specifically consent to

that. However, the law does not require that any adverse events that you may suffer are documented and reported.

**Overview of Study Procedures**

| | Screening Period | Blood for Method Validation | Study Period | | | Safety Follow Period |
|---|---|---|---|---|---|---|
| **Study Visit** | **Screening** | | **Visit 1** | **Visit 2** | **Visit 3** | |
| **Week** | | | **1** | **2** | **3** | **3** |
| Procedure | | | | | | |
| Informed consent | X | | | | | |
| Demographics | X | | | | | |
| Medical history | X | | | | | |
| Vital signs | X | | X | X | X | |
| Hematology | X | | | | | |
| Clinical chemistry | X | | | | | |
| Urinalysis | X | | | | | |
| Urine pregnancy test (If applicable) | X | | | | | |
| Concomitant medication | X | | X | X | X | |
| Physical examination | X | | | | | |
| PK plasma sampling | | | X | X | X | |
| Urine sampling | | | X | X | X | |
| Study drug administration | | | X | X | X | |
| Safety monitoring | | | X | X | X | |
| Reimbursement | | | | | | X |
| Telephone call | | | | | | X |
