# Supplementary material for: Pharmacokinetics and Metabolomic Profiling of Metformin and Andrographis paniculata: A Protocol for a Crossover Randomised Controlled Trial
Source: J Clin Med. 2022 Jul 6;11(14):3931. doi: 10.3390/jcm11143931 (PMC9323336; doi:10.3390/jcm11143931)
Supplement: Supplementary file 1 [file jcm-11-03931-s001.zip › jcm-1697297-supplementary.pdf]

Contents

Figure S1. Ethics committee approval letter.. 2

Table S1. Trial activities schedule 3

Figure S2. Randomization methods 4

2.1 Randomization table.. 4

2.2 Randomization source code 4

Figure S3. Investigational product dispensing activity record.. 5

Figure S4. Representative investigational product label 6

Figure S5. Subject investigational product dosing time 7

Figure S6. Subject blood sampling time... 8

Figure S7. List of tables, listing and figures... 9

Figure S8. Workflow for Metabolomic Data Processing and Statistical Analysis. 10

Figure S1. Ethics committee approval letter

UNIVERSITY OF MALAYA MEDICAL CENTRE

MEDICAL RESEARCH ETHICS COMMITTEE
(Formerly known as Medical Ethics Committee)
UNIVERSITY OF MALAYA MEDICAL CENTRE
ADDRESS : LEMBAH PANTAI, 59100 KUALA LUMPUR, MALAYSIA
TELEPHONE : 03-79493209/2251 FAXIMILE : 03-79492030

**NAME OF ETHICS COMMITTEE/IRB**<br>Medical Research Ethics Committee, University Malaya Medical Centre	**MREC ID NO:** 2018112-6848
**ADDRESS :** LEMBAH PANTAI, 59100 KUALA LUMPUR, MALAYSIA	
**PROTOCOL NO**(if applicable) : P1-PKPD-Metabolomic	
**TITLE:**<br>A Phase 1, Open Label, Randomized, Three-Period, Crossover, Single Dose Oral Administration Of Andrographis Paniculata And Metformin Clinical Trial In Healthy Volunteers Under Fasting Condition.	
**PRINCIPAL INVESTIGATOR :** LUQMAN BIN IBRAHIM	**SPONSOR**<br>Bantuan Kecil Penyelidikan Universiti Malaya

The following item [✓] have been received and reviewed in connection with the above study to conducted by the above investigator.

[✓] Application for Amendment/Notification to Research Project (form)	Ver.No : -	Ver.Date : 29-07-2019
[ ] Annual Study Report/Study Closure Report	Ver.No : -	Ver.Date : -
[ ] Serious Adverse Event Report	Ver.No : -	Ver.Date : -
[✓] Other documents		
1) GCP certificate Dr Luqman bin Ibrahim	Ver.No : 2.0	Ver.Date : 16-07-2019
2) Revised Clinical Trial Insurance	Ver.No : 2.0	Ver.Date : 05-11-2018
3) Clinical Trial Protocol P1-PKPD-Metabolomic	Ver.No : 2.0	Ver.Date : 16-07-2019
4) P1-PKPD-Metabolomic Patient Information Sheet English	Ver.No : 2.0	Ver.Date : 16-07-2019
5) P1-PKPD-Metabolomic Patient Information Sheet Malay	Ver.No : 2.0	Ver.Date : 16-07-2019
6) P1-PKPD-Metabolomic Informed Consent Form English	Ver.No : 2.0	Ver.Date : 16-07-2019
7) P1-PKPD-Metabolomic Informed Consent Form Malay	Ver.No : 2.0	Ver.Date : 16-07-2019
8) P1-PKPD-Metabolomic Advertisement	Ver.No : 2.0	Ver.Date : 16-07-2019
9) Curriculum vitae Dr Luqman Ibrahim	Ver.No : 1.0	Ver.Date : 23-07-2019

and the decision is [✓]

[✓] Approved (Expedited)
[ ] Approved (Full Board)
[ ] Rejected (reasons specified below or in accompanying letter)
[ ] Noted

Comments:

-

The investigators are required to:

1) *follow instructions, guidelines and requirements of the Medical Research Ethics Committee.*
2) *report any protocol deviations/violations to Medical Research Ethics Committee.*
3) *provide annual and closure report to the Medical Research Ethics Committee.*
4) *comply with International Conference on Harmonization – Guidelines for Good Clinical Practice (ICH-GCP) and Declaration of Helsinki.*
5) *obtain a permission from the Director of UMMC to start research that involves recruitment of UMMC patient.*
6) *ensure that if the research is sponsored, the usage of consumable items and laboratory tests from UMMC services are not charged in the patient's hospital bills but are borne by research grant.*
7) *note that he/she can appeal to the Chairman of Medical Research Ethics Committee for studies that are rejected.*
8) *note that Medical Research Ethics Committee may audit the approved study.*
9) *ensure that the study does not take precedence over the safety of subjects.*

Date of expedited approval : 01-08-2019
Date of notification : -
This is a computer generated letter. No signature required.

Table S1. Trial activities schedule

Protocol: P1-PKPD-Metabolomic

Activities Schedule

Study Phase	Screening	MV Sampling	Period 1	Ambulatory	Period 2	Ambulatory	Period 3	Ambulatory	Safety Follow-up
Attendance List	√	√	√	√	√	√	√	√	
Informed Consent	√								
Demographics	√								
Medical History	√								
Physical Examination	√		√		√		√		
Clinical Assessment	√		√		√		√		
ECG (12-lead)	√								
Vital signs Measurement	√		√		√		√		
Hematology	√							√	
Urinalysis	√							√	
Serum Chemistry	√							√	
Urine Drug Screening	√								
HIV/Hep B&C	√								
MV Blood and urine samplings		√							
Drug dosing			√		√		√		
PK Blood sampling			√	√	√	√	√	√	
Urine sampling			√		√		√		
Glucose monitoring			√		√		√		
AE Monitoring			√		√		√		
Phone call follow up									√

Figure S2. Randomization methods

2.1 Randomization table

Seed number = 736522

block	subject	sequence
1	001	A B C
1	002	C A B
1	003	B C A
1	004	C A B
1	005	B C A
1	006	A B C
2	001	A B C
2	002	C A B
2	003	B C A
2	004	C A B
2	005	A B C
2	006	B C A
3	001	C A B
3	002	C A B
3	003	B C A
3	004	A B C
3	005	B C A
3	006	A B C

2.2 Randomization source code

SAS Randomization Program Code Seed 736522

```
data _null_;
x=round(ranuni(0)*1000000);
call symput ('seed', x);title "Seed number = &seed.";
run;
title "Seed number = &seed.";
proc plan seed=&seed.;
factors block=3 ordered subject=6 ordered/noprint;
treatments sequence=6 random;
output out=out
 sequence cvals=('A B C' 'A B C' 'B C A' 'B C A' 'C A B' 'C A B');
run;
proc print data=out noobs;
var block subject sequence ;
format subject z3.;
run;#
```

Figure S3. Investigational product dispensing activity record

Protocol No: P1-PKPD-Metabolomic

Investigational Product Dispensing Activity

Date:

Investigational product:

No	Steps	Performed by
1	Clean the dispensing table with alcohol or ensure the table is clean and empty.	
2	Prepare the __ labels, spoon, envelops.	
3	Check the randomization table seed no: list with labels.	
4	Stick the label on each envelope.	
5	Retrieve the IP time:	
6	Put __ capsules/tablets into the envelope with label and seal the envelope.	
7	Check the envelopes with randomization table seed no:	
8	Return the IP time:	
9	Put the finish IP into container.	

Investigational Product Dispensing Activity

Date:

Investigational product:

No	Steps	Performed by
1	Clean the dispensing table with alcohol or ensure the table is clean and empty.	
2	Prepare the __ labels, spoon, envelops.	
3	Check the randomization table seed no: list with labels	
4	Stick the label on each envelope.	
5	Retrieve the IP time:	
6	Put __ capsules/tablets into the envelope with label and seal the envelope.	
7	Check the envelopes with randomization table seed no:	
8	Return the IP time:	
9	Put the finish IP into container.	

Figure S4. Representative investigational product label

"FOR CLINICAL TRIAL PURPOSE ONLY"				"FOR CLINICAL TRIAL PURPOSE ONLY"			
Protocol No: P1-PKPD-Metabolomics		S:1	P:1	Protocol No: P1-PKPD-Metabolomics		S:1	P:1
Generic Name:	Metformin 1000mg			Generic Name:	Metformin 1000mg		
Route of Administration	oral	Manufactured by:	Merck Sante s.a.s	Route of Administration	oral	Manufactured by:	Merck Sante s.a.s
Storage Condition:	Store below 30ºC	Batch/Lot No:	Y04655	Storage Condition:	Store below 30ºC	Batch/Lot No:	Y04655
Quantity:	2 tablets	Expiry date:	10/2023	Quantity:	2 tablets	Expiry date:	10/2023
Dispensing date	23.9.19	Dosing date		Dispensing date	23.9.19	Dosing date	
"FOR CLINICAL TRIAL PURPOSE ONLY"				"FOR CLINICAL TRIAL PURPOSE ONLY"			
Protocol No: P1-PKPD-Metabolomics		S:6	P:1	Protocol No: P1-PKPD-Metabolomics		S:6	P:1
Generic Name:	Metformin 1000mg			Generic Name:	Metformin 1000mg		
Route of Administration	oral	Manufactured by:	Merck Sante s.a.s	Route of Administration	oral	Manufactured by:	Merck Sante s.a.s
Storage Condition:	Store below 30ºC	Batch/Lot No:	Y04655	Storage Condition:	Store below 30ºC	Batch/Lot No:	Y04655
Quantity:	2 tablets	Expiry date:	10/2023	Quantity:	2 tablets	Expiry date:	10/2023
Dispensing date	23.9.19	Dosing date		Dispensing date	23.9.19	Dosing date	
"FOR CLINICAL TRIAL PURPOSE ONLY"				"FOR CLINICAL TRIAL PURPOSE ONLY"			
Protocol No: P1-PKPD-Metabolomics		S:7	P:1	Protocol No: P1-PKPD-Metabolomics		S:7	P:1
Generic Name:	Metformin 1000mg			Generic Name:	Metformin 1000mg		
Route of Administration	oral	Manufactured by:	Merck Sante s.a.s	Route of Administration	oral	Manufactured by:	Merck Sante s.a.s
Storage Condition:	Store below 30ºC	Batch/Lot No:	Y04655	Storage Condition:	Store below 30ºC	Batch/Lot No:	Y04655
Quantity:	2 tablets	Expiry date:	10/2023	Quantity:	2 tablets	Expiry date:	10/2023
Dispensing date	23.9.19	Dosing date		Dispensing date	23.9.19	Dosing date	
"FOR CLINICAL TRIAL PURPOSE ONLY"				"FOR CLINICAL TRIAL PURPOSE ONLY"			
Protocol No: P1-PKPD-Metabolomics		S:2	P:2	Protocol No: P1-PKPD-Metabolomics		S:2	P:2
Generic Name:	Metformin 1000mg			Generic Name:	Metformin 1000mg		
Route of Administration	oral	Manufactured by:	Merck Sante s.a.s	Route of Administration	oral	Manufactured by:	Merck Sante s.a.s
Storage Condition:	Store below 30ºC	Batch/Lot No:	Y04655	Storage Condition:	Store below 30ºC	Batch/Lot No:	Y04655
Quantity:	2 tablets	Expiry date:	10/2023	Quantity:	2 tablets	Expiry date:	10/2023
Dispensing date	23.9.19	Dosing date		Dispensing date	23.9.19	Dosing date	
"FOR CLINICAL TRIAL PURPOSE ONLY"				"FOR CLINICAL TRIAL PURPOSE ONLY"			
Protocol No: P1-PKPD-Metabolomics		S:4	P:2	Protocol No: P1-PKPD-Metabolomics		S:4	P:2
Generic Name:	Metformin 1000mg			Generic Name:	Metformin 1000mg		
Route of Administration	oral	Manufactured by:	Merck Sante s.a.s	Route of Administration	oral	Manufactured by:	Merck Sante s.a.s
Storage Condition:	Store below 30ºC	Batch/Lot No:	Y04655	Storage Condition:	Store below 30ºC	Batch/Lot No:	Y04655
Quantity:	2 tablets	Expiry date:	10/2023	Quantity:	2 tablets	Expiry date:	10/2023
Dispensing date	23.9.19	Dosing date		Dispensing date	23.9.19	Dosing date	
"FOR CLINICAL TRIAL PURPOSE ONLY"				"FOR CLINICAL TRIAL PURPOSE ONLY"			
Protocol No: P1-PKPD-Metabolomics		S:8	P:2	Protocol No: P1-PKPD-Metabolomics		S:8	P:2
Generic Name:	Metformin 1000mg			Generic Name:	Metformin 1000mg		
Route of Administration	oral	Manufactured by:	Merck Sante s.a.s	Route of Administration	oral	Manufactured by:	Merck Sante s.a.s
Storage Condition:	Store below 30ºC	Batch/Lot No:	Y04655	Storage Condition:	Store below 30ºC	Batch/Lot No:	Y04655
Quantity:	2 tablets	Expiry date:	10/2023	Quantity:	2 tablets	Expiry date:	10/2023
Dispensing date	23.9.19	Dosing date		Dispensing date	23.9.19	Dosing date	
"FOR CLINICAL TRIAL PURPOSE ONLY"				"FOR CLINICAL TRIAL PURPOSE ONLY"			
Protocol No: P1-PKPD-Metabolomics		S:3	P:3	Protocol No: P1-PKPD-Metabolomics		S:3	P:3
Generic Name:	Metformin 1000mg			Generic Name:	Metformin 1000mg		
Route of Administration	oral	Manufactured by:	Merck Sante s.a.s	Route of Administration	oral	Manufactured by:	Merck Sante s.a.s
Storage Condition:	Store below 30ºC	Batch/Lot No:	Y04655	Storage Condition:	Store below 30ºC	Batch/Lot No:	Y04655
Quantity:	2 tablets	Expiry date:	10/2023	Quantity:	2 tablets	Expiry date:	10/2023
Dispensing date	23.9.19	Dosing date		Dispensing date	23.9.19	Dosing date	
"FOR CLINICAL TRIAL PURPOSE ONLY"				"FOR CLINICAL TRIAL PURPOSE ONLY"			
Protocol No: P1-PKPD-Metabolomics		S:5	P:3	Protocol No: P1-PKPD-Metabolomics		S:5	P:3
Generic Name:	Metformin 1000mg			Generic Name:	Metformin 1000mg		
Route of Administration	oral	Manufactured by:	Merck Sante s.a.s	Route of Administration	oral	Manufactured by:	Merck Sante s.a.s
Storage Condition:	Store below 30ºC	Batch/Lot No:	Y04655	Storage Condition:	Store below 30ºC	Batch/Lot No:	Y04655
Quantity:	2 tablets	Expiry date:	10/2023	Quantity:	2 tablets	Expiry date:	10/2023
Dispensing date	23.9.19	Dosing date		Dispensing date	23.9.19	Dosing date	
"FOR CLINICAL TRIAL PURPOSE ONLY"				"FOR CLINICAL TRIAL PURPOSE ONLY"			
Protocol No: P1-PKPD-Metabolomics		S:9	P:3	Protocol No: P1-PKPD-Metabolomics		S:9	P:3
Generic Name:	Metformin 1000mg			Generic Name:	Metformin 1000mg		
Route of Administration	oral	Manufactured by:	Merck Sante s.a.s	Route of Administration	oral	Manufactured by:	Merck Sante s.a.s
Storage Condition:	Store below 30ºC	Batch/Lot No:	Y04655	Storage Condition:	Store below 30ºC	Batch/Lot No:	Y04655
Quantity:	2 tablets	Expiry date:	10/2023	Quantity:	2 tablets	Expiry date:	10/2023
Dispensing date	23.9.19	Dosing date		Dispensing date	23.9.19	Dosing date	

Figure S5. Subject investigational product dosing time

Subject IP Dosing Time

Date:__________ Period: __________

Dosing Procedure: [Print 3 pages for cohort 1]

1. Checked the identity tag.
2. Checked the label.
3. Dose the subject with the IP with a glass of water.
4. Mouth checked after complete dosing.
5. Write the dosing time and sign.

Subject ID	T0	Actual Dosing Time	Subject Signature	SC signature
**S1**	8.00			
**S2**	8.02			
**S3**	8.04			
**S4**	8.06			
**S5**	8.08			
**S6**	8.00			
**S7**	8.02			
**S8**	8.04			
**S9**	8.06			

Figure S6. Subject blood sampling time

Protocol No: P1-PKPD-Metabolomics

Subject Blood Sampling Time

Subject ID	T0	T0.5	T1	T1.5	T2	T2.5	T3.0	T3.5	T4	T5	T6	T8	T10	T12	T24
**S1**	8.00	8.30	9.00	9.30	10.00	10.30	11.00	11.30	12.00	13.00	14.00	16.00	18.00	20.00	8.00
**S2**	8.02	8.32	9.02	9.32	10.02	10.32	11.02	11.32	12.02	13.02	14.02	16.02	18.02	20.02	8.02
**S3**	8.04	8.34	9.04	9.34	10.04	10.34	11.04	11.34	12.04	13.04	14.04	16.04	18.04	20.04	8.04
**S4**	8.06	8.36	9.06	9.36	10.06	10.36	11.06	11.36	12.06	13.06	14.06	16.06	18.06	20.06	8.06
**S5**	8.08	8.38	9.08	9.38	10.08	10.38	11.08	11.38	12.08	13.08	14.08	16.08	18.08	20.08	8.08
**S6**	8.00	8.30	9.00	9.30	10.00	10.30	11.00	11.30	12.00	13.00	14.00	16.00	18.00	20.00	8.00
**S7**	8.02	8.32	9.02	9.32	10.02	10.32	11.02	11.32	12.02	13.02	14.02	16.02	18.02	20.02	8.02
**S8**	8.04	8.34	9.04	9.34	10.04	10.34	11.04	11.34	12.04	13.04	14.04	16.04	18.04	20.04	8.04
**S9**	8.06	8.36	9.06	9.36	10.06	10.36	11.06	11.36	12.06	13.06	14.06	16.06	18.06	20.06	8.06
**S10**	8.00	8.30	9.00	9.30	10.00	10.30	11.00	11.30	12.00	13.00	14.00	16.00	18.00	20.00	8.00
**S11**	8.02	8.32	9.02	9.32	10.02	10.32	11.02	11.32	12.02	13.02	14.02	16.02	18.02	20.02	8.02
**S12**	8.04	8.34	9.04	9.34	10.04	10.34	11.04	11.34	12.04	13.04	14.04	16.04	18.04	20.04	8.04
**S13**	8.06	8.36	9.06	9.36	10.06	10.36	11.06	11.36	12.06	13.06	14.06	16.06	18.06	20.06	8.06
**S14**	8.08	8.38	9.08	9.38	10.08	10.38	11.08	11.38	12.08	13.08	14.08	16.08	18.08	20.08	8.08
**S15**	8.00	8.30	9.00	9.30	10.00	10.30	11.00	11.30	12.00	13.00	14.00	16.00	18.00	20.00	8.00
**S16**	8.02	8.32	9.02	9.32	10.02	10.32	11.02	11.32	12.02	13.02	14.02	16.02	18.02	20.02	8.02
**S17**	8.04	8.34	9.04	9.34	10.04	10.34	11.04	11.34	12.04	13.04	14.04	16.04	18.04	20.04	8.04
**S18**	8.06	8.36	9.06	9.36	10.06	10.36	11.06	11.36	12.06	13.06	14.06	16.06	18.06	20.06	8.06

Figure S7. List of tables, listing and figures.

1. Clinical part
   A. Table for subject demographic and clinical laboratory data
   B. List of adverse events
   C. Listing of vital sign monitoring

2. Pharmacokinetics

   Method validation

   i. Listing of accuracy, precision, calibration curve, stability and others.

   Subject sample analysis and in-study validation

   A. *Andrographis paniculata* 1000mg and 2000mg for three bioactive compounds; andrographolide, neoandrographolide and deoxyandrographolide

      i. Table: Plasma concentration time-point
      ii. Figures: Plasma concentration time-point/ AUC
      iii. Table: Maximum plasma concentration, time to reach the maximum plasma concentration, volume of distribution, clearance and half-life.

   B. Metformin 1000mg

      i. Table: Plasma concentration time-point
      ii. Figures: Plasma concentration time-point/ AUC
      iii. Table: Maximum plasma concentration, time to reach the maximum plasma concentration, volume of distribution, clearance and half-life.

3. Untargeted metabolomics for plasma and urine samples
   A. Metformin 1000mg, *Andrographis paniculata* 1000mg and 2000mg
      i. Figure: principal component analysis and/or partial least square discriminant analysis for metabolites in pre-dose versus peak plasma post-dose.
      ii. Listing: Predicted perturbed human metabolic pathways between time-point pre-dose and peak plasma post dose.
      iii. Figure: Identification of the significant pathway from Kyoto Encyclopedia of Gene and Genome

Figure S8. Workflow for Metabolomic Data Processing and Statistical Analysis.

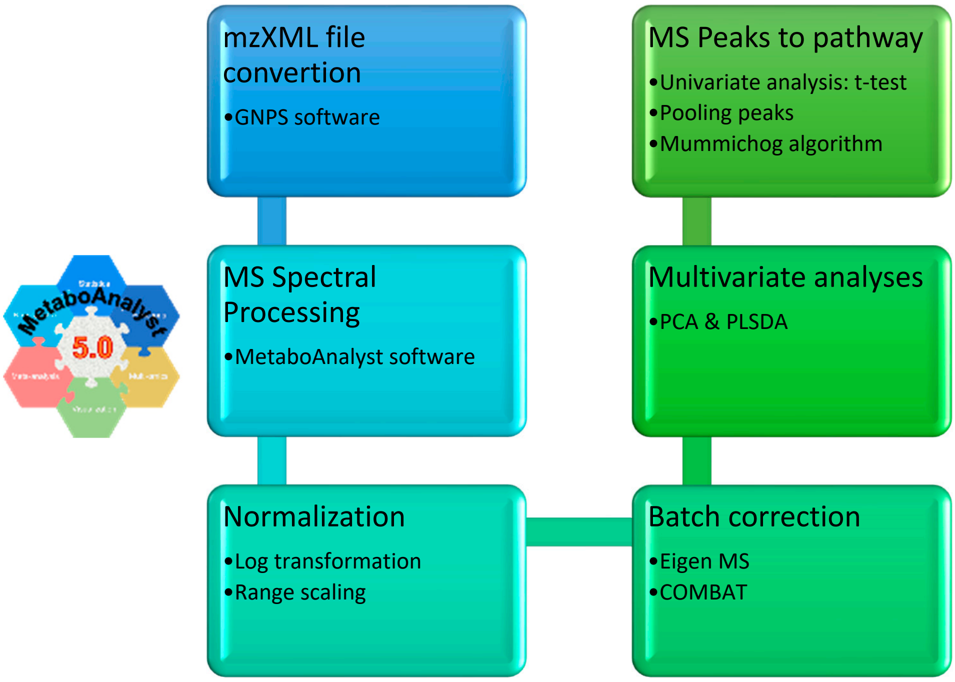

1. Convert the chromatograms from .d file to mzXML file using GNPS software.
2. Perform the MS Spectral Processing module using batch data of subject samples and pool quality control samples in MetaboAnalyst software to obtain list of metabolites according to subject samples.
3. Perform normalization statistical analysis for the batch data in MetaboAnalyst software.
4. Continue the batch correction when the samples were run in different days or instrument interruption using Eigen MS or COMBAT analysis.
5. Run multivariate analyses using the principal component analysis (PCA) and partial least square discriminant analysis (PLSDA) with cross validation using MetaboAnalyst software or SIMCA software.
6. Using the MS Peaks to pathway module in the MetaboAnalyst software. The module included t-test to identify significant features, follow by pooling peaks and to match the significant features with Kyoto Encyclopedia of Gene and Genome (KEGG) to predict the dysregulated human metabolic pathways using mummichog algorithm.
7. Above step 1 to 7 workflow will be repeated for *Andrographis paniculata* 1000mg, *Andrographis paniculata* 2000mg and Metformin 1000mg.
